# Supplementary material for: Inclusion of participants from low-income and middle-income countries in core outcome sets development: a systematic review
Source: BMJ Open. 2021 Oct 18;11(10):e049981. doi: 10.1136/bmjopen-2021-049981 (PMC8527127; doi:10.1136/bmjopen-2021-049981)
Supplement: Supplementary data [file bmjopen-2021-049981supp001.pdf]

Appendix A. Search strategy (As described by Gorst et al [1]

**Search terms for MEDLINE**	
	**Randomised trial and systematic review terms**
1	Health Services/ut [Utilization]
2	registries/
3	systematic review.mp.
4	structured review.ti.
5	evidence based medicine.ab.
6	exp Clinical Trials as Topic/
7	clinical trial$.ab.
8	randomised controlled trial$.ti,ab.
9	randomised trial$.ti,ab.
10	1 or 2 or 3 or 4 or 5 or 6 or 7 or 8 or 9
	**Methodology terms**
11	workgroup$.mp.
12	standard$ outcome$.mp.
13	Practice Guideline/
14	clinical database.mp.
15	patient important outcome$.mp.
16	(standard$ adj3 reporting).mp.
17	congresses.pt.
18	Delphi Technique/
19	(recommend$ adj3 outcome$).mp.
20	consensus development conference.pt.
21	outcome$ reporting.mp.
22	priorit$ symptom$.mp.
23	(task force adj3 outcome$).mp.
24	appropriate outcome$.mp.
25	research design/
26	endpoint determination/
27	consensus development conference/
28	patient participation/
29	consensus.mp.
30	workshop.mp.
31	Consensus Development Conferences, NIH as Topic/
32	focus groups/
33	11 or 12 or 13 or 14 or 15 or 16 or 17 or 18 or 19 or 20 or 21 or 22 or 23 or 24 or 25 or 26 or 27 or 28 or 29 or 30 or 31 or 32
	**Outcome terms**
34	outcome$.mp.

Karumbi J, *et al. BMJ Open* 2021; 11:e049981. doi: 10.1136/bmjopen-2021-049981

35	end point$.mp.
36	(core adj3 set).mp.
37	treatment emergent problem$.mp.
38	exp outcome Assessment Health Care/
39	Treatment Outcome/
40	Quality of Life/
41	34 or 35 or 36 or 37 or 38 or 39 or 40
	**Key terms targeted**
42	clinical-study design.mp.
43	patient$ perspective$.ti.
44	outcome$.mp. and delphi.ti.
45	(outcome$ and delphi).ab.
46	(perspective$ adj3 outcome$).ti.
47	core outcome$.ti,ab.
48	core set$.ti,ab.
49	clinical trial design$.ti.
50	design$ clinical trial$.ti.
51	(consensus and outcome$).ti.
52	42 or 43 or 44 or 45 or 46 or 47 or 48 or 49 or 50 or 51
53	10 and 33 and 41
54	52 or 53

**Search terms for SCOPUS**
((((INDEXTERMS(registries)) OR (INDEXTERMS(clinical trials as topic)) OR (ABS("evidence based medicine")) OR (ABS("clinical trial*")) OR (INDEXTERMS("Health Services Utilization")) OR (TITLE-ABS-KEY("SYSTEMATIC REVIEW")) OR (TITLE("structured review"))) OR (TITLE OR ABS("randomised controlled trial*")) OR (TITLE OR ABS (randomised trial*))) AND (((TITLE-ABS-KEY(workgroup*)) OR (TITLE-ABS-KEY(standard* outcome*)) OR (INDEXTERMS(practice guideline)) OR (TITLE-ABS-KEY("clinical database")) OR (TITLE-ABS-KEY("patient important outcome*")) OR (TITLE-ABS-KEY("standard* outcome*")) OR (INDEXTERMS(delphi technique))) OR ((TITLE-ABS-KEY(recommend* W/3 outcome*)) OR (TITLE-ABS-KEY(standard* W/3 reporting*)) OR (TITLE-ABS-KEY(task force W/3 outcome*)) OR (TITLE-ABS-KEY("appropriate outcome*")) OR (TITLE-ABS-KEY("outcome* reporting")) OR (TITLE-ABS-KEY("priorit* symptom*")) OR (INDEXTERMS(focus group)) (INDEXTERMS(research design))) OR ((INDEXTERMS(endpoint determination)) OR (INDEXTERMS(consensus development conference)) OR (INDEXTERMS(patient participation)) OR (TITLE-ABS-KEY(consensus)) OR (TITLE-ABS-KEY(workshop)))) AND 74) OR (((TITLE("design* clinical trials")) OR (TITLE(consensus AND outcome*)) OR (TITLE-ABS-KEY("clinical-study design")) OR (TITLE("patient* perspective*")) OR (ABS(outcome* AND delphi)) OR (TITLE(outcome* AND delphi)) OR (TITLE(perspective* W/3 outcome*)) OR (ABS("core outcome*") OR TITLE("core outcome*"))) OR ((ABS("core set*") OR TITLE("core set*")) OR (TITLE("clinical trial design*"))))

[1] Gorst SL, Prinsen CAC, Salcher-Konrad M, Matvienko-Sikar K, Williamson PR, Terwee CB. Methods used in the selection of instruments for outcomes included in core outcome sets have improved since the publication of the COSMIN/COMET guideline. J Clin Epidemiol 2020;125:64–75. https://doi.org/https://doi.org/10.1016/j.jclinepi.2020.05.021.
