# Supplementary material for: Inclusion of participants from low-income and middle-income countries in core outcome sets development: a systematic review
Source: BMJ Open. 2021 Oct 18;11(10):e049981. doi: 10.1136/bmjopen-2021-049981 (PMC8527127; doi:10.1136/bmjopen-2021-049981)
Supplement: Author's manuscript [file bmjopen-2021-049981.draft_revisions.pdf]

BMJ Open is committed to open peer review. As part of this commitment we make the peer review history of every article we publish publicly available.

When an article is published we post the peer reviewers' comments and the authors' responses online. We also post the versions of the paper that were used during peer review. These are the versions that the peer review comments apply to.

The versions of the paper that follow are the versions that were submitted during the peer review process. They are not the versions of record or the final published versions. They should not be cited or distributed as the published version of this manuscript.

BMJ Open is an open access journal and the full, final, typeset and author-corrected version of record of the manuscript is available on our site with no access controls, subscription charges or pay-per-view fees (http://bmjopen.bmj.com).

If you have any questions on BMJ Open's open peer review process please email info.bmjopen@bmj.com

BMJ Open

Inclusion of participants from LMICs in Core Outcome Sets development: a systematic review

Journal:	*BMJ Open*
Manuscript ID	bmjopen-2021-049981
Article Type:	Original research
Date Submitted by the Author:	09-Feb-2021
Complete List of Authors:	Karumbi, Jamlick; University of Liverpool, Health Data Science; KEMRI-Wellcome Trust Research Programme Nairobi, Health Systems Research<br>Gorst, Sarah L.; University of Liverpool, Department of Health Data Science<br>Gathara, David; Kenya Medical Research Institute, Wellcome Trust Research Program ; London School of Hygiene & Tropical Medicine<br>Gargon, Elizabeth; University of Liverpool, Department of Health Data Science<br>Young, Bridget; University of Liverpool, Department of Health Data Science<br>Williamson, Paula; University of Liverpool, Health Data Science
Keywords:	EPIDEMIOLOGY, PUBLIC HEALTH, Protocols & guidelines < HEALTH SERVICES ADMINISTRATION & MANAGEMENT

SCHOLARONE™
Manuscripts

I, the Submitting Author has the right to grant and does grant on behalf of all authors of the Work (as defined in the below author licence), an exclusive licence and/or a non-exclusive licence for contributions from authors who are: i) UK Crown employees; ii) where BMJ has agreed a CC-BY licence shall apply, and/or iii) in accordance with the terms applicable for US Federal Government officers or employees acting as part of their official duties; on a worldwide, perpetual, irrevocable, royalty-free basis to BMJ Publishing Group Ltd ("BMJ") its licensees and where the relevant Journal is co-owned by BMJ to the co-owners of the Journal, to publish the Work in this journal and any other BMJ products and to exploit all rights, as set out in our licence.

The Submitting Author accepts and understands that any supply made under these terms is made by BMJ to the Submitting Author unless you are acting as an employee on behalf of your employer or a postgraduate student of an affiliated institution which is paying any applicable article publishing charge ("APC") for Open Access articles. Where the Submitting Author wishes to make the Work available on an Open Access basis (and intends to pay the relevant APC), the terms of reuse of such Open Access shall be governed by a Creative Commons licence – details of these licences and which Creative Commons licence will apply to this Work are set out in our licence referred to above.

Other than as permitted in any relevant BMJ Author's Self Archiving Policies, I confirm this Work has not been accepted for publication elsewhere, is not being considered for publication elsewhere and does not duplicate material already published. I confirm all authors consent to publication of this Work and authorise the granting of this licence.

For peer review only

Inclusion of participants from LMICs in Core Outcome Sets development: a systematic review

Karumbi Jamlick[1,2,3*], Sarah Gorst[1], David Gathara [3,4], Elizabeth Gargon[1], Bridget Young[1], Paula Williamson[1]

[1] Department of Health Data Science, University of Liverpool

[2] Ministry of Health, Kenya

[3] Kemri-Welcome Trust Research Programme

[4] London School of Hygiene and Tropical Medicine

* Correspondence: J.Karumbi@liverpool.ac.uk

Abstract

Objective: Our study aims to describe differences or similarities in the scope, participant characteristics and methods used in Core Outcome Sets (COS) development when only participants from high income countries (HICs) were involved compared to when participants from low- and middle-income countries (LMICs) were also involved.

Design/setting: This is a review of published studies.

Methods: Studies were eligible for inclusion if they developed or applied methodology for determining which outcome domains or outcomes should be measured and were listed in the Core Outcome Measures in Effectiveness Trials (COMET) database. Data were extracted in relation to scope of the COS study, participant characteristics and the methods used in outcome selection.

Results: Studies describing 370 COS were identified in the database. Of these, 75 (20%) included participants from LMICs. Only four COS were initiated from an LMIC setting. More than half of COS with LMIC participants were developed in the last 5 years. Cancer and rheumatology were the dominant disease domains. Overall, over 259 (70%) of COS explicitly reported including clinical experts; this was higher where LMIC participants were also included 340 (92%). Most LMIC participants were from China, Brazil, and South Africa. Mixed methods for consensus building were used across the two settings.

Conclusion: Progress has been made in including LMIC participants in the development of COS, however there is a need to explore how to enable initiation of COS development from a range of LMIC settings, how to ensure prioritization of COS that better reflects the burden of disease in these contexts and how to improve public participation from LMICs.

For peer review only - http://bmjopen.bmj.com/site/about/guidelines.xhtml

Strengths and limitations of this study

- This is the first paper describing the differences and similarities in the COS development processes when there are participants from LMICs compared to when participants are exclusively from HIC settings.
- This paper describes COS published up to 2019. It is possible that more COS involving LMICs participants have been developed during 2020 or are in the process of being developed now.
- Public participation has been described in only a few studies, this may introduce a possible bias in the classification of public participants categories thereby altering the conclusions of public participation

Background

A key barrier to translating clinical research into practice is lack of conclusive evidence. This is due to different trials, of the similar interventions, being too heterogenous and reporting on outcomes that are often non comparable [1,2] or not relevant to patients and users [3,4].

Development of core outcome sets (COS) could ensure all future research in a field reports a common subset of outcomes. This would reduce research waste thereby enhancing comparability and improving research translation and use[5,6]. COS are agreed-on minimum standardized outcome sets that should be measured and reported in all research in a given health area[7]. They consist of a core domain set (this defines what domains should be measured) and core outcome measurement set (defines the instruments which would be appropriate to measure the domain)[8]. In recent years, the use of COS has been promoted by journals, Cochrane review groups, and funders[9,10]. COS have also become useful in routine clinical practice data collection for clinical audit and feedback[11,12].

Since 2010, the Core Outcome Measures in Effectiveness Trials (COMET) Initiative has been collating, stimulating and promoting the development and use of COS by maintaining an up to date publicly available searchable database[13]. The COMET database includes published studies of COS development, as well as planned and ongoing work. Initially, the database included studies that had been identified by ad hoc means but has since become systematic in the update of the included COS with annually updated systematic reviews being the basis of the database updates since 2013[14–17]. The use of this database should help minimize duplication of effort in the development of COS and potentially encourage the use of COS to tackle global health challenges.

How are COS developed?

There is no gold standard for development of COS, however minimum standards have been agreed in the last few years. These are the Core Outcome Set-STAndards for Development

For peer review only - http://bmjopen.bmj.com/site/about/guidelines.xhtml

(COS-STAD) recommendations, comprising 11 minimum standards for all COS development projects[18]. The recommendations focus on three key domains: (i) the scope of the COS; specific area of health or healthcare that the COS applies needs to be described, with details of health condition, population and types of interventions. The COS may be developed to encompass all stages or severity of a health condition or it may be focused on a particular disease category. (ii) the stakeholders involved in the COS development; the stakeholder groups to be involved and the target number from each group is dependent upon the scope of the COS and the existing knowledge and practicability. Decisions on stakeholders' involvement should be documented and explained in the study protocol. (iii) the consensus processes; this typically involves developing a consensus on 'what', 'how' and 'when' to measure. Some of the methods used to build consensus include: Delphi technique; nominal group technique, consensus development conference and semi structured group discussion. The Delphi technique with two or three rounds combined with a consensus conference has been most widely used[17,19,20]. Choice of method is informed by several factors including; the need to build a true consensus with methodological rigor, strategies to ensure that a diverse range of opinions are heard, and factors such as financial and carbon costs that might limit the practicality of face-to-face meetings[7].

To date, COS have been developed for various thematic or disease conditions and more are being developed[13,17]. It has been opined that development and use of a COS would help reduce outcome heterogeneity and reporting bias, while ensuring that wide-ranging perspectives, including patients' opinions, are incorporated, thus enhancing the value and quality of research[21]. Most COS have been developed from the perspectives of high-income countries (HICs), with over 70% of COS including participants from Europe and North America. There has been an increase in the proportion of COS that have included participants from Africa and South American countries in COS development, but this still remains low at around 25% as per COS published up to the end of 2018 [17]. A closer look shows that the proportion of COS participants residing in low- and middle-income countries (LMICs) of these regions is even lower being just about 16% in 2016. Further, participants have mainly been those with research and clinical expertise in the various disease areas rather than as public participants with relevant lived experience [16].

Previous work describing COS studies [14–17] has not examined whether there is a difference in the scope, stakeholder participation and approaches to consensus between COS that have

For peer review only - http://bmjopen.bmj.com/site/about/guidelines.xhtml

include had participants only from HICs compared to those that also included participants from LMICs. Given the differences in the global burden of diseases, which can potentially influence which COS are developed, and resources availability which can potentially determine the methods used in COS development, it is important to examine the differences/similarities across these two settings. The aim of this paper is to describe the differences or similarities in the scope, participant characteristics and methods used in COS developed with participants from HICs and LMICs.[1].

Methods

Study selection

Inclusion criteria: Studies were eligible for inclusion if they developed or applied methodology for determining which outcome domains or outcomes should be measured in research.

Identification of relevant studies: We identified any studies that had been published before the end of 2019 in the COMET database. The process of including studies in the COMET Initiative database has previously been described [14–17] and is briefly described here. Studies are eligible for inclusion if they have applied methodology for determining which outcome domains or outcomes should be measured in clinical trials or other forms of health research. Studies are eligible for inclusion if they reported the development of a COS, regardless of any restrictions by age, health condition or setting. Studies describing the development of a patient reported outcome (PRO) COS (a core set of patients reported symptoms and health-related quality of life domains) or core event set (a core set of adverse events or complications) are also eligible for inclusion. Studies describing the update of an existing COS are included as linked papers to the original COS[17]. Eligible studies are added to the database, as they are found with the annual update to the systematic review, ensuring the database is kept up to date.

Data extraction:

[1]https://datahelpdesk.worldbank.org/knowledgebase/articles/906519-world-bank-country-and-lending-groups). Accessed on 20th April 2020

For peer review only - http://bmjopen.bmj.com/site/about/guidelines.xhtml

Data were extracted in relation to scope of the COS study [aim of the study, intended use of the COS, year of publication, target population, intervention and disease or condition area], participant categories [clinicians, researchers etc, and their geographic location] and the methods used in outcome selection [Delphi, interviews, focus group discussions, etc, alone or in combination]. Data on the burden of disease in LMICs for the last five years was obtained, from the Institute of Health Metrics and Evaluation database[22], to help check its alignment to the COS developed over the same period.

Analysis:

A description of the proportion of COS that included participants from LMICs and those which were initiated from the LMIC setting is provided. For a COS to be classified as being initiated from an LMIC, either ethical approval of the study had to have been sought in an LMIC, and/or the affiliation of the lead author had to be based in an LMIC; we also used the sites of participant recruitment as confirmation. The scope, participant categories and consensus methods used are described for COS that included participants from LMICs and those with exclusively HIC participants separately. Participation here is defined as being a study participant and contributing data to the COS study.

Findings

General description

There was a total of 552 references published by end of 2019 in the COMET Initiative database describing 370 COS. Of these 370 COS, 295 had only HIC participants and 75 (20%) included LMIC participants. Two COS had only LMIC participants, one in Sri Lanka [23] and another in China [24]. A flow diagram of the study selection process is provided in Figure 1 as guided by Preferred Reporting Items for Systematic Reviews and Meta-Analyses statement[25].

Year of publication

There has been an almost two-fold increase in COS that have included participants from LMICs in the last five years [15% (35/229) of COS developed up to 2014 compared to 28% (40/141) of COS developed between 2014 and 2019]. Figure 2 describes the absolute numbers of the COS developed up to 2019.

For peer review only - http://bmjopen.bmj.com/site/about/guidelines.xhtml

Scope of included studies

The comparison of the scope of the included studies is shown in Table 1. Overall, more than half (63%) of the studies were specifically designed for COS development rather than being part of a wider project or trial. However, a higher proportion of studies with LMIC participants were specific for COS (83%) compared to those with only HIC participants (58%). Over 80% of COS were developed for research only. More than half of studies (55%) did not explicitly report on the age group of the target population characteristics although it was assumed that in the absence of explicit mention the target population was adults, not children.

Table 1 Scope of included studies

	HICs n (%) (N=295)	LMICs# n (%) (N=75)	Total
**Scope of the COS study**			
**Study aims**			
Part of wider trial design	124 (42)	13 (17)	137 (37)
Specific for COS	171 (58)	62 (83)	233 (63)
**Intended use of recommendations**			
Research	264 (89)	61 (81)	325 (88)
Research and Practice	31 (11)	14 (19)	45 (12)
**Population characteristics**			
Neonates	4 (1)	1 (1)	5 (1)
Adults	72 (24)	16 (21)	88 (24)
Children	27 (9)	7 (9)	34 (9)
Children and Adults	28 (9)	10 (13)	38 (10.3)
Not specified*	164 (56)	41 (55)	205 (55)
**Intervention characteristic**			
Any intervention	77 (26)	30 (40)	107 (29)
Device	5 (2)	1 (1)	6 (2)
Device & Surgery	7 (2)	1 (1)	8 (2)
Pharmacologic Treatment	50 (17)	9 (12)	59 (16)
Procedure	9 (3)	5 (7)	14 (4)
Radiotherapy	2 (1)	0 (0)	2 (1)
Rehabilitation	9 (3)	2 (3)	11 (3)
Screening	2 (1)	0 (0)	2 (1)
Surgical	31 (11)	6 (8)	37 (10)
Vaccine	0 (0)	2 (3)	2 (3)
Both Pharmacologic & Pharmacologic treatment	3 (1)	0 (0)	3 (1)
Non-Pharmacologic Treatment	4 (1)	2 (3)	6 (2)
Health System	5 (2)	2 (3)	7 (2)
Not specified	91 (31)	15 (20)	106 (29)

** assumed to mean adults*

Cancer and rheumatology were the dominant disease domains across the COS developed either with LMIC or HIC participants. A slightly higher proportion of COS for rheumatology, pregnancy, skin, anaesthesia, and child health included participants from LMICs compared to HICs. Five COS on health systems had LMIC participants. Figure 3 describes the disease categories that were covered by the COS.

For peer review only - http://bmjopen.bmj.com/site/about/guidelines.xhtml

Of the 75 COS with LMIC participants, only 5% were initiated from LMIC setting. All were from Asia, with two from China[24,26] and one each from Sri-Lanka[23] and the Philippines[27]. No COS has been initiated from Africa or South America, although COS for conditions which are more prevalent in LMIC settings had a higher number of participants from LMICs. For example, the malaria COS by Moorthy et al [28] included participants from ten sub-Sahara African Countries but none from other LMICs. The COS for the prevention of preterm births had LMIC participants from Egypt, Nigeria, South Africa, China, Iran, Lebanon, Pakistan, Argentina and Brazil[29].

Characteristics of participants involved in consensus process

All COS that had LMIC participants explicitly reported characteristics of their sample compared to 92% of COS that had only HIC participants (see Table 2). Overall, over 70% of COS explicitly reported including clinical experts; this was higher where LMIC participants were involved (92%). A slightly higher proportion of COS that included LMIC participants (57%) had included members of the public compared to those with HIC participants only (32%). A higher proportion of LMIC COS (23%) had carers as part of public participants compared to the HIC COS (9%). Of the 28 COS with LMIC participants, only 4 (all with HIC patient participants too) translated the COS development materials to non-English languages. These are COS for clinical trials in acute diarrhoea [30], infant colic [31], childhood constipation [32] and type 2 diabetes[33]. Non-clinical research expertise was more common in COS with LMIC participants (44%) compared to those with HIC participants only (31%).

Table 2: Participant characteristics

Participant category	Sub category (not mutually exclusive)	HIC n (%#)	LMIC n (%#)	Total (%#)
**Clinical experts**		**201/295 (68)**	**69/75 (92)**	**270/370 (73)**
	Clinical experts	166 (56)	62 (83)	228 (62)
	Clinical research experts	92 (31)	41 (55)	133 (36)
	Members of a clinical trial network	8 (3)	7 (9)	15 (4)
	Clinical and non-clinical researchers	12 (4)	1 (1)	13 (4)
**Public participation**		**94/295 (32)**	**43/75 (57)**	**137/370 (37)**
	Patients	72 (24)	28 (37)	100 (27)
	Carers	26 (9)	17 (23)	43 (12)
	Patient support group representatives	21 (7)	9 (12)	30 (8)
	Service users	4 (1)	5 (7)	9 (2)
**Non-clinical research expertise**		**92/295 (31)**	**33/75 (44)**	**125/370 (34)**
	Researchers	55 (19)	21 (28)	76 (21)
	Statisticians	22 (7)	8 (11)	30 (8)
	Epidemiologists	13 (4)	9 (12)	22 (6)

For peer review only - http://bmjopen.bmj.com/site/about/guidelines.xhtml

	Academic representatives	5 (2)	0 (0)	5 (1)
	Methodologists	19 (6)	5 (7)	24 (6)
	Economists	7 (2)	2 (3)	9 (2)
**Authorities**		**50/295 (17)**	**22/75 (29)**	**72/370 (19)**
	Regulatory agency representatives	33 (11)	15 (20)	48 (13)
	Government agencies	14 (5)	5 (7)	19 (5)
	Policy makers	10 (3)	7 (9)	17 (5)
	Charities	4 (1)	0 (0)	4 (1)
	Service commissioners	3 (1)	1 (1)	4 (1)
**Industry representatives**		**36/295 (12)**	**19/75 (25)**	**55/270 (20)**
	Pharmaceutical industries	32 (11)	19 (25)	51 (14)
	Device manufacturers	4 (1)	1 (1)	5 (1)
	Biotech company representatives	1 (1)	0 (0)	1 (1)
**Others**		**10/295 (3)**	**8/75 (11)**	**18/370 (5)**
	Service providers	5 (2)	2 (3)	7 (2)
	Ethicists	1 (1)	1 (1)	2 (1)
	Journal editors	4 (1)	5 (7)	9 (2)
	Systematic review authors	13 (4)	2 (3)	15 (4)
**Not stated**	Not reported	23 (8)	0 (0)	23 (6)

[#] Since the categories are not mutually exclusive each percentage is based on the total for that category, i.e. 295 for HIC, 75 for LMIC and 370 for the Total.

Participants from LMICs were drawn from the three continents of Asia (60%), South America (49%) and Africa (31%). However, there was a skewed representation with LMIC participants largely being from three countries, with South Africa representing 50% of participants from Africa, Brazil representing 46% of participants from South America and China representing 30% of the participants from Asia.

Methods for selection of outcomes

Methods used for outcome selection ranged from Delphi, consensus conferences and semi structured discussion as shown in Table 3 below. Rarely was one method used for COS development. Semi-structured discussion seemed to be the most commonly used single method, with 20% of COS with HIC participants only and 8% of COS with LMIC participants describing its usage.

Most COS used a combination of methods. Literature review in combination with other methods was most common, with 37% of COS with HIC participants and 60% of COS with LMIC participants using this method as shown in Table 3 below. Only one COS that included LMIC participants had no methods described compared to six that only had HIC participants.

For peer review only

For peer review only - http://bmjopen.bmj.com/site/about/guidelines.xhtml

Table 3: Methods used in the selection of outcomes

Description	HIC n=295 (%)	LMIC n=75 (%)	Total n=370 (%)
Delphi only	10 (3)	5 (7)	15 (4)
Consensus conference only	11 (4)	4 (5)	15 (4)
Focus group discussions only	1 (<1)	0 (0)	1 (<1)
Nominal Group Techniques only	1 (<1)	0 (0)	1 (<1)
Semi structured discussions only	64 (22)	6 (8)	70 (19)
Survey only	4 (1)	1 (1)	5 (1)
Review of literature only	32 (4)	4 (5)	36 (10)
Interviews only	1 (<1)	0 (0)	1 (<1)
**Mixed methods**	**171/295 (58)**	**55/75 (73)**	**226/370 (61)**
Consensus conference and other(s)	8(3)	1 (1)	9 (2)
Delphi and other(s)	73(25)	42 (56)	115 (31)
Focus group discussions and other(s)	7 (2)	7 (9)	14 (4)
Nominal Group Techniques and other(s)	15 (5)	11 (15)	26 (7)
Semi structured discussions and other(s)	88 (30)	33 (44)	121 (33)
Survey and other(s)	25 (8)	8 (11)	33 (9)
Review of literature and other(s)	108 (37)	45 (60)	153 (41)
Interviews and other(s)	20 (7)	7 (9)	27 (7)
Unstructured and other(s)	4 (1)	8 (11)	12 (3)
No methods described	6 (2)	1 (1)	7 (2)

A combination of the three most common methods was more likely to be used for LMIC COS (n=23, 30%) compared to the HIC COS (n=37, 13%) as shown in Figure 4.

Discussion

This review provides the first comparison of COS that have been developed with participants from HICs and LMICs.

Out of the 370 published COS, 75 (20%) COS included participants from LMICs. Thirty-three COS have been published since the fifth update of COS systematic review with fifteen including participants from LMICs [17]. The COS span a range of health areas, target populations and methods used. The methods for outcome selection are similar across the settings, perhaps reflecting the increased standardization of methods of COS development generally.

Interestingly, even though participants from LMICs have been involved in earlier COS, for example, the cancer reporting recommendations by Miller and others in 1981 [34], the inclusion of LMIC participants has increased predominantly in the last 5 years. In this time, there has been an almost two-fold increase in the proportion of COS that have had LMIC participants. Global connectivity is allowing people to conduct research without the need for

For peer review only - http://bmjopen.bmj.com/site/about/guidelines.xhtml

physical meetings. A look at the development processes used is 'older' COS shows that most depended on consensus building during conferences and physical workshops [28,34,35]. These kinds of consensus meetings tend to be more expensive and limit inclusion of participants from varied settings. Despite the increase, Brazil, China and South Africa have been the main drivers of participation from LMICs.

All the COS developed across the settings have mainly focused on research or research and practice. A slightly higher proportion (19%) of COS with LMIC participants are for research and practice compared to 11% of COS with HIC participants only. Given that COS with participants from LMICs are 'newer' this could reflect that more recent COS are being developed with the intention of spanning the continuum from research to routine practice in mind.

Although the COS span a wide range of conditions, the top five conditions where COS exist are cancer, rheumatology, neurology, cardiovascular and orthopaedic conditions. These are mainly non communicable diseases, and even though these are now becoming more common in LMICs, the burden of these conditions has mainly been in HICs [22]. Of the COS with LMIC participants, pregnancy, lungs and health systems were among the top five conditions. LMICs are still facing infectious diseases like neonatal sepsis, lower respiratory tract infections, malaria, and HIV among others[22,36], and one would therefore expect that COS with LMIC participation would reflect this. Only two COS, for malaria [28] and leprosy [27] represent conditions that are usually of high burden in LMICs. Thus, even though LMIC participants are being included in the COS development, COS may not be reflecting the disease burden in LMICs.

Interestingly, only four COS have been initiated from an LMIC setting, two from China (COS for hip-preserving treatment of osteonecrosis of the femoral head [24], COS for infertility treatment research [26]) and one each in Sri-Lanka (COS for effectiveness of antiepileptic therapy in children [23]) and the Philippines, (COS for tibialis posterior transfer surgery [27]). There is still no COS initiated from Africa or South America. Given the varying disease epidemiology and burden between HICs and LMICs there is a need to identify mechanisms to enable LMIC stakeholders to develop context relevant COS so as to improve applicability and adoptability of COS to these settings [14]. These strategies could probably include awareness raising about COS in LMIC settings, provision of resources for COS development and dissemination of guidelines and methods for COS development. These are some of the barriers which have been described potentially affecting COS development from a HIC

For peer review only

perspective (interviewees were mainly from Europe and North America) [37]. There is a need to assess which barriers and enablers are particularly relevant to COS development in LMIC settings.

Details of public participation are still sub-optimally reported, and where reported, a higher proportion of COS with LMIC participants had some public participation compared to COS with HIC participants only. Of note however, is that public participants tended to be from HIC settings. For example, the Type 2 diabetes COS included public participants from the UK and Greece[33], the breast cancer COS included breast cancer patients from US [38] and the COS for acute diarrhoea [30], infant colic [31], childhood constipation [32] all included public participants from Europe. Public participation from LMICs could potentially be improved by translation of consensus building materials into non-English languages. Harman et al, translated Delphi materials into Portuguese language in a bid to improve public participation from Brazil [33]. Unfortunately, there still were no public participants from Brazil suggesting that there are additional reasons for the lack of public participants from LMIC settings.

The lack of public participations from LMICs could be due to numerous reasons some of which could include: temporal, cultural and resources differences between LMICs and HICs. First, COS that have included participants from LMICs have mostly been developed in the last decade, during which guidance has been published on how to develop COS including patient participation. Second, the availability of organized patient groups in HIC settings for given conditions or diseases provides a means of identifying and inviting patients as public participants; such groups are generally lacking in LMIC settings. Third, information asymmetry between the public and patients versus clinicians or researchers could limit the participation of the former in COS development[39]. Additionally, some of the major research funders in HICs, for example the National Institute for Health Research in the UK and the Patient-Centred Outcomes Research Institute in the US, are increasingly championing public participation in research.

It is therefore likely that a multipronged approach is needed. Further research to explore what would work, in addition to language translation, to improve public participation from LMICs is also needed.

Additionally, there is need to describe the stage at which public participants are involved in COS development and whether it influences which outcomes are included in the COS in a given area. Previous work has shown that patients might sometimes be inhibited when put

For peer review only - http://bmjopen.bmj.com/site/about/guidelines.xhtml

together with clinicians in a consensus building process[40] but recent experiences show this is changing, with high satisfaction reported, at least from HIC countries, where joint consensus meetings are held[33,41].

This is the first paper describing the differences and similarities in the COS development processes when there are participants from LMICs compared to when participants are exclusively from HIC settings. However, there are a few limitations. First, this paper describes COS published up to 2019. It is possible that more COS involving LMICs participants have been developed during 2020 or are in the process of being developed now. Secondly, public participation has been described in only a few studies, this may introduce a possible bias in the classification of public participants categories thereby altering the conclusions of public participation.

Conclusion

Progress has been made in including LMIC participants in the development of COS, however there is a need to explore how to enable initiation of COS development from a range of LMIC settings, how to ensure prioritization of COS that better reflects the burden of disease in these contexts and how to improve public participation from LMICs.

ACKNOWLEDGEMENTS

This work is also published with the permission of the Director of KEMRI.

COMPETING INTERESTS

None declared

AUTHORS CONTRIBUTIONS

The roles of the contributors were as follows: JK, DG, BY, SG, EG and PW conceptualized the review. JK screened all title and abstracts supported by PW and SG who carried out double screening of these articles. Full-text articles were screened by JK and any uncertainties were discussed with the team. JK and SG extracted data. JK drafted the initial manuscript. All authors reviewed the draft manuscript and approved the final manuscript

For peer review only - http://bmjopen.bmj.com/site/about/guidelines.xhtml

FUNDING

JK has been supported to undertake this work as part of PhD studentship from the University of Liverpool. This studentship has no award number. The University of Liverpool had no role in developing this manuscript nor in the decision to submit for publication.

PATIENTS AND PUBLIC INVOLVEMENT

Patients and the public were not involved in the development of the research question or the design and conduct of this systematic review.

For peer review only

References

[1] Heneghan C, Goldacre B, Mahtani KR. Why clinical trial outcomes fail to translate into benefits for patients. Trials 2017;18:122. https://doi.org/10.1186/s13063-017-1870-2.

[2] Duffy JMN, Ziebland S, von Dadelszen P, McManus RJ. Tackling poorly selected, collected, and reported outcomes in obstetrics and gynecology research. Am J Obstet Gynecol 2019;220:71.e1-71.e4. https://doi.org/10.1016/j.ajog.2018.09.023.

[3] Sinha IP, Williamson PR, Smyth RL. Outcomes in Clinical Trials of Inhaled Corticosteroids for Children with Asthma Are Narrowly Focussed on Short Term Disease Activity. PLoS One 2009;4:e6276.

[4] Tong A, Crowe S, Gill JS, Harris T, Hemmelgarn BR, Manns B, et al. Clinicians' and researchers' perspectives on establishing and implementing core outcomes in haemodialysis: semistructured interview study. BMJ Open 2018;8:e021198. https://doi.org/10.1136/bmjopen-2017-021198.

[5] Clarke M. Standardising outcomes for clinical trials and systematic reviews. Trials 2007;8:1–3. https://doi.org/10.1186/1745-6215-8-39.

[6] Chalmers I, Glasziou P. Avoidable waste in the production and reporting of research evidence. Lancet (London, England) 2009;374:86–9. https://doi.org/10.1016/S0140-6736(09)60329-9.

[7] Williamson PR, Altman DG, Blazeby JM, Clarke M, Devane D, Gargon E, et al. Developing core outcome sets for clinical trials: issues to consider. Trials 2012;13:132. https://doi.org/10.1186/1745-6215-13-132.

[8] Chevance A, Tran VT, Ravaud P. Improving the generalizability and credibility of core outcome sets (COSs) by a large and international participation of diverse stakeholders. J Clin Epidemiol 2020:1–8. https://doi.org/10.1016/j.jclinepi.2020.01.004.

[9] Hughes KL, Kirkham JJ, Clarke M, Williamson PR. Assessing the impact of a research funder's recommendation to consider core outcome sets. PLoS One 2019;14:e0222418–e0222418. https://doi.org/10.1371/journal.pone.0222418.

[10] COMET Initiative. COS endorsments 2020. http://www.comet-initiative.org/COSEndorsement (accessed September 26, 2020).

[11] Williamson PR, Altman DG, Bagley H, Barnes KL, Blazeby JM, Brookes ST, et al. The COMET Handbook: version 1.0. Trials 2017;18:280. https://doi.org/10.1186/s13063-017-1978-4.

[12] Webbe JWH, Duffy JMN, Afonso E, Al-Muzaffar I, Brunton G, Greenough A, et al. Core outcomes in neonatology: development of a core outcome set for neonatal research. Arch Dis Child - Fetal Neonatal Ed 2019:fetalneonatal-2019-317501. https://doi.org/10.1136/archdischild-2019-317501.

[13] COMET Initiative. COMET_database n.d. http://www.comet-initiative.org/Studies (accessed February 10, 2020).

[14] Gargon E, Gurung B, Medley N, Altman DG, Blazeby JM, Clarke M, et al. Choosing important health outcomes for comparative effectiveness research: A systematic review. PLoS One 2014;9. https://doi.org/10.1371/journal.pone.0099111.

[15] Gorst SL, Gargon E, Clarke M, Blazeby JM, Altman DG, Williamson PR. Choosing important health outcomes for comparative effectiveness research: An updated review and user survey. PLoS One 2016;11:1–12. https://doi.org/10.1371/journal.pone.0146444.

[16] Davis K, Gorst SL, Harman N, Smith V, Gargon E, Altman DG, et al. Choosing important health outcomes for comparative effectiveness research: An updated systematic review and involvement of low and middle income countries. PLoS One 2018;13:1–14. https://doi.org/10.1371/journal.pone.0190695.

[17] Gargon E, Gorst SL, Williamson PR. Choosing important health outcomes for comparative effectiveness research: 5th annual update to a systematic review of core outcome sets for research. PLoS One 2019;14:e0225980–e0225980. https://doi.org/10.1371/journal.pone.0225980.

[18] Kirkham JJ, Davis K, Altman DG, Blazeby JM, Clarke M, Tunis S, et al. Core Outcome Set-

For peer review only - http://bmjopen.bmj.com/site/about/guidelines.xhtml

STAndards for Development: The COS-STAD recommendations. PLoS Med 2017;14:e1002447–e1002447. https://doi.org/10.1371/journal.pmed.1002447.
[19] Sinha IP, Smyth RL, Williamson PR. Using the Delphi technique to determine which outcomes to measure in clinical trials: recommendations for the future based on a systematic review of existing studies. PLoS Med 2011;8:e1000393–e1000393. https://doi.org/10.1371/journal.pmed.1000393.
[20] Turnbull AE, Dinglas VD, Friedman LA, Chessare CM, Sepúlveda KA, Bingham III CO, et al. A survey of Delphi panelists after core outcome set development revealed positive feedback and methods to facilitate panel member participation. J Clin Epidemiol 2018;102:99–106. https://doi.org/10.1016/j.jclinepi.2018.06.007.
[21] Williamson P, Altman D, Blazeby J, Clarke M, Gargon E. Driving up the Quality and Relevance of Research Through the Use of Agreed Core Outcomes. J Health Serv Res Policy 2012;17:1–2. https://doi.org/10.1258/jhsrp.2011.011131.
[22] Institute of Health Metrics and Evaluation. Global Burden of Disease 2020. https://vizhub.healthdata.org/gbd-compare/ (accessed December 1, 2020).
[23] Murugupillai R, Ranganathan SS, Wanigasinghe J, Muniyandi R, Arambepola C. Development of outcome criteria to measure effectiveness of antiepileptic therapy in children. Epilepsy Behav 2018;80:56–60. https://doi.org/10.1016/j.yebeh.2017.12.011.
[24] Xue Z, Sun J, Li T, Huang Z, Chen W. How to evaluate the clinical outcome of joint-preserving treatment for osteonecrosis of the femoral head: development of a core outcome set. J Orthop Surg Res 2019;14:317. https://doi.org/10.1186/s13018-019-1364-x.
[25] Moher D, Liberati A, Tetzlaff J, Altman DG, Group TP. Preferred Reporting Items for Systematic Reviews and Meta-Analyses: The PRISMA Statement. PLOS Med 2009;6:e1000097.
[26] Legro RS, Wu X, Barnhart KT, Farquhar C, Fauser BCJM, Mol B. Improving the reporting of Clinical trials of infertility treatments (IMPRINT): Modifying the CONSORT statement. Hum Reprod 2014;29:2075–82. https://doi.org/10.1093/humrep/deu218.
[27] Cross H. A Delphi consensus on criteria for contraindications, assessment indicators and expected outcomes related to tibialis posterior transfer surgery. Int J Lepr Other Mycobact Dis Off Organ Int Lepr Assoc 2005;73:13–21. https://doi.org/10.1489/1544-581X(2005)73[13:ADCOCF]2.0.CO;2.
[28] Moorthy V, Reed Z, Smith PG. Measurement of malaria vaccine efficacy in phase III trials: Report of a WHO consultation. Vaccine 2007;25:5115–23. https://doi.org/10.1016/j.vaccine.2007.01.085.
[29] Van't Hooft J, Duffy JMN, Daly M, Williamson PR, Meher S, Thom E, et al. A core outcome set for evaluation of interventions to prevent preterm birth. Obstet Gynecol 2016;127:49–58. https://doi.org/10.1097/AOG.0000000000001195.
[30] Karas J, Ashkenazi S, Guarino A, Vecchio A Lo, Shamir R, Vandenplas Y, et al. A core outcome set for clinical trials in acute diarrhoea. Arch Dis Child 2015;100:359–63. https://doi.org/10.1136/archdischild-2014-307403.
[31] Steutel NF, Benninga MA, Langendam MW, Korterink JJ, Indrio F, Szajewska H, et al. Developing a core outcome set for infant colic for primary, secondary and tertiary care settings: A prospective study. BMJ Open 2017;7:1–11. https://doi.org/10.1136/bmjopen-2016-015418.
[32] Kuizenga-Wessel S, Steutel NF, Benninga MA, Devreker T, Scarpato E, Staiano A, et al. Development of a core outcome set for clinical trials in childhood constipation: a study using a Delphi technique. BMJ Paediatr Open 2017;1:e000017. https://doi.org/10.1136/bmjpo-2017-000017.
[33] Harman NL, Wilding JPH, Curry D, Harris J, Logue J, Pemberton RJ, et al. Selecting Core Outcomes for Randomised Effectiveness trials In Type 2 diabetes (SCORE-IT): a patient and healthcare professional consensus on a core outcome set for type 2 diabetes. BMJ Open Diabetes Res &Amp; Care 2019;7:e000700. https://doi.org/10.1136/bmjdrc-2019-

For peer review only - http://bmjopen.bmj.com/site/about/guidelines.xhtml

000700.
[34] Miller A, Hoogstraten B, Staquet M, Winkler A. Reporting Results of Cancer Trials. Cancer 1981;47:207–14.
[35] Heijde V der D, Bellamy N, Calin A. Preliminary Core Sets for Endpoints in Ankylosing Spondilitis. J Rheumatol 1997;24:2225–9.
[36] Vos T, Lim SS, Abbafati C, Abbas KM, Abbasi M, Abbasifard M, et al. Global burden of 369 diseases and injuries in 204 countries and territories, 1990–2019: a systematic analysis for the Global Burden of Disease Study 2019. Lancet 2020;396:1204–22. https://doi.org/10.1016/S0140-6736(20)30925-9.
[37] Gargon E, Williamson PR, Young B. Improving core outcome set development: qualitative interviews with developers provided pointers to inform guidance. J Clin Epidemiol 2017;86:140–52. https://doi.org/10.1016/j.jclinepi.2017.04.024.
[38] Ong WL, Schouwenburg MG, Van Bommel ACM, Stowell C, Allison KH, Benn KE, et al. A standard set of value-based patient-centered outcomes for breast cancer: The International Consortium for Health Outcomes Measurement (ICHOM) initiative. JAMA Oncol 2017;3:677–85. https://doi.org/10.1001/jamaoncol.2016.4851.
[39] Bloom G, Standing H, Lloyd R. Markets, information asymmetry and health care: Towards new social contracts. Soc Sci Med 2008;66:2076–87. https://doi.org/10.1016/j.socscimed.2008.01.034.
[40] Young B, Bagley H. Including patients in core outcome set development: issues to consider based on three workshops with around 100 international delegates. Res Involv Engagem 2016;2:25. https://doi.org/10.1186/s40900-016-0039-6.
[41] Hall DA, Smith H, Hibbert A, Colley V, Haider HF, Horobin A, et al. The COMiT'ID Study: Developing Core Outcome Domains Sets for Clinical Trials of Sound-, Psychology-, and Pharmacology-Based Interventions for Chronic Subjective Tinnitus in Adults. Trends Hear 2018;22:1–16. https://doi.org/10.1177/2331216518814384.

List of figures

Figure 1 Preferred Reporting Items for Systematic Reviews and Meta-Analyses flowchart of identification of eligible studies from the COMET database. Data were extracted from the COS systematic reviews [14–17]. 5
Figure 2 Year of COS publication 6
Figure 3 Comparison of disease categories covered in the COS with exclusively HIC participants and those also with LMIC participants 7
Figure 4 Combinations of methods for outcome selection 9

For peer review only - http://bmjopen.bmj.com/site/about/guidelines.xhtml

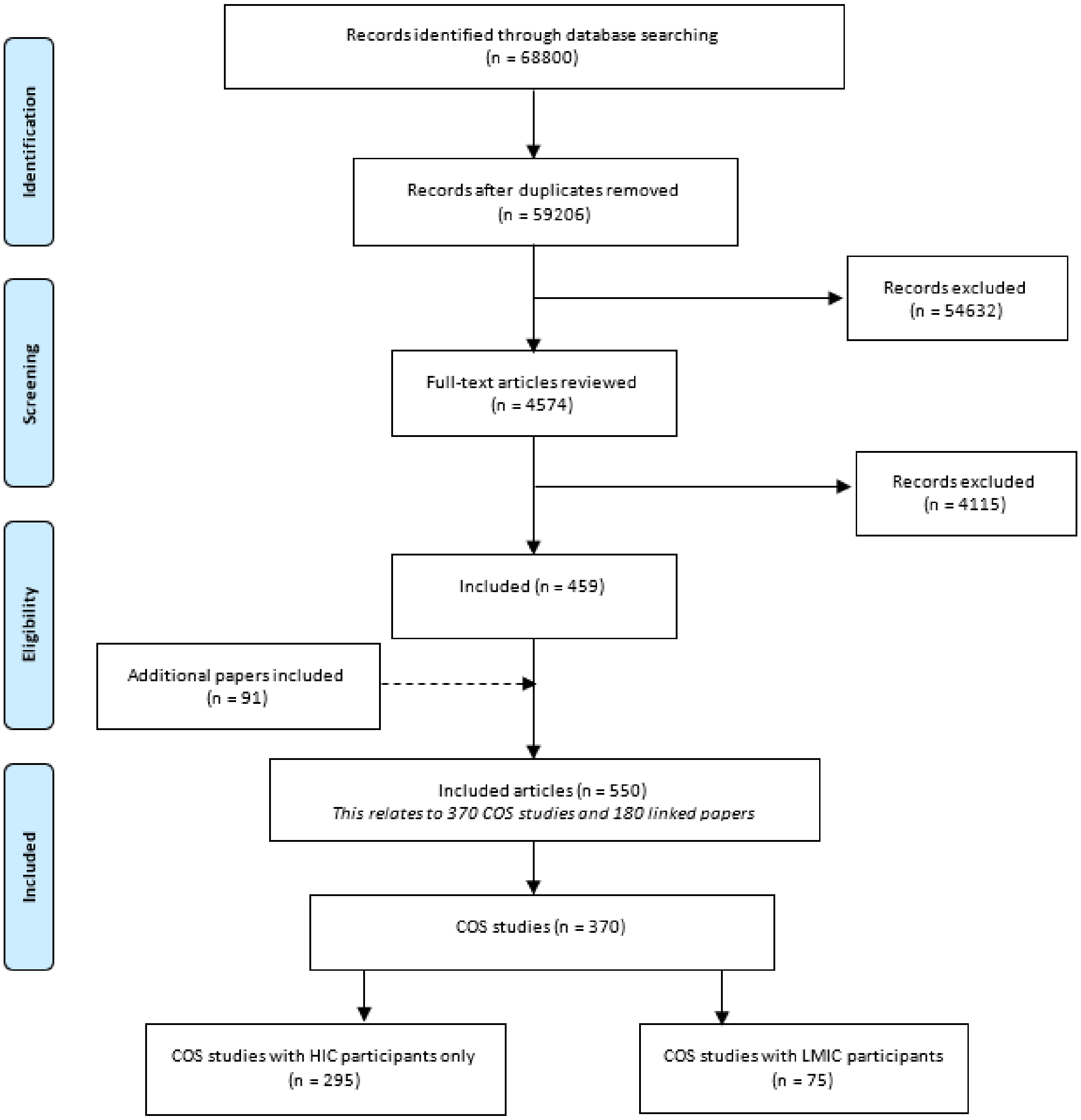

Figure 1 Preferred Reporting Items for Systematic Reviews and Meta-Analyses flowchart of identification of eligible studies from the COMET database. Data were extracted from the COS systematic reviews [14–17].

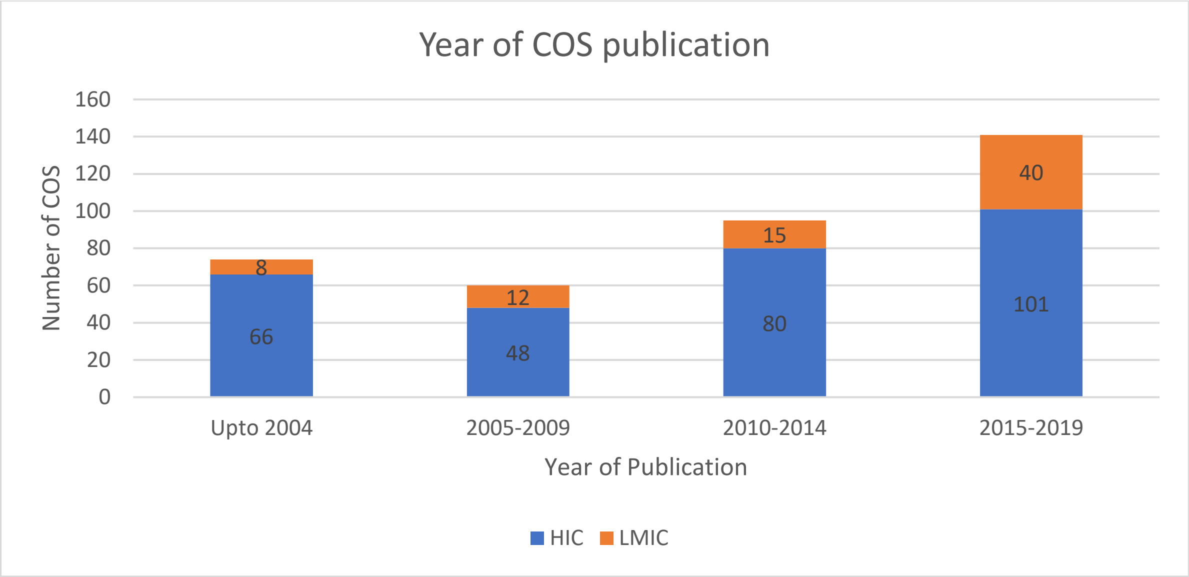

Figure 2 Year of COS publication

For peer review only

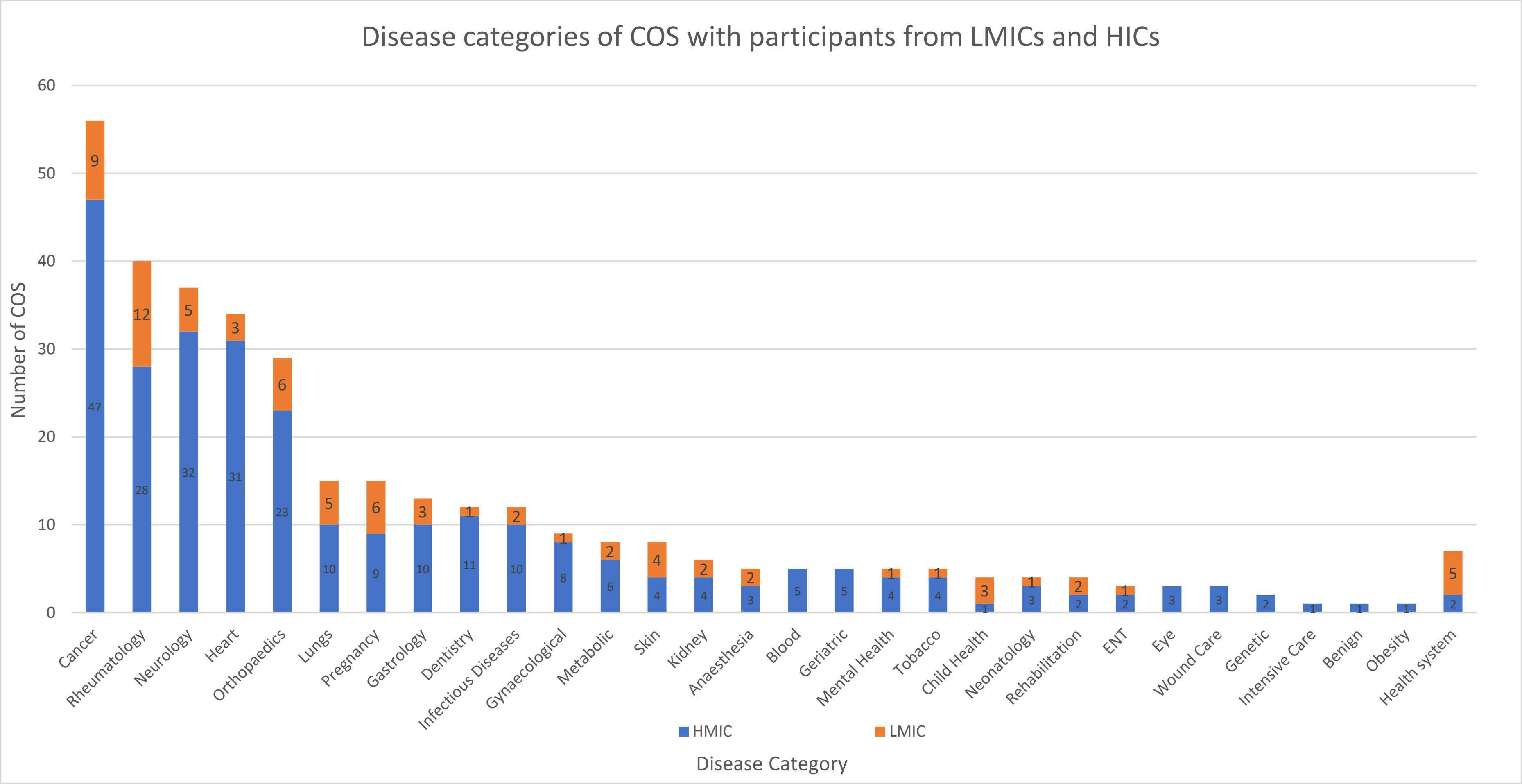

Figure 3 Comparison of disease categories covered in the COS with exclusively HIC participants and those also with LMIC participants

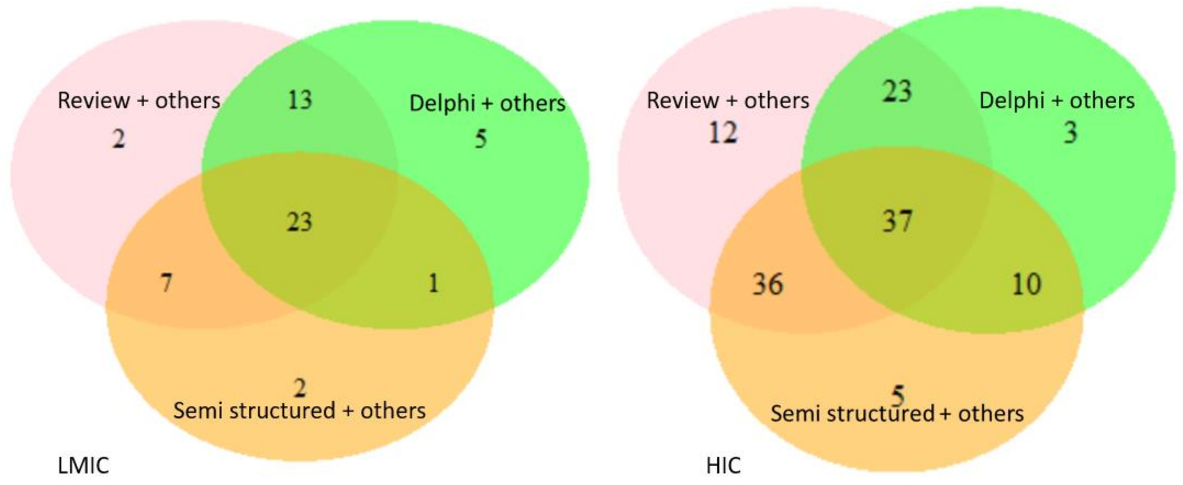

Figure 4 Methods used in COS development across LMIC and HIC

PRISMA 2009 Checklist

Section/topic	#	Checklist item	Reported on page #
**TITLE**			
Title	1	Identify the report as a systematic review, meta-analysis, or both.	1
**ABSTRACT**			
Structured summary	2	Provide a structured summary including, as applicable: background; objectives; data sources; study eligibility criteria, participants, and interventions; study appraisal and synthesis methods; results; limitations; conclusions and implications of key findings; systematic review registration number.	1
**INTRODUCTION**			
Rationale	3	Describe the rationale for the review in the context of what is already known.	3
Objectives	4	Provide an explicit statement of questions being addressed with reference to participants, interventions, comparisons, outcomes, and study design (PICOS).	5
**METHODS**			
Protocol and registration	5	Indicate if a review protocol exists, if and where it can be accessed (e.g., Web address), and, if available, provide registration information including registration number.	none
Eligibility criteria	6	Specify study characteristics (e.g., PICOS, length of follow-up) and report characteristics (e.g., years considered, language, publication status) used as criteria for eligibility, giving rationale.	5
Information sources	7	Describe all information sources (e.g., databases with dates of coverage, contact with study authors to identify additional studies) in the search and date last searched.	5
Search	8	Present full electronic search strategy for at least one database, including any limits used, such that it could be repeated.	5
Study selection	9	State the process for selecting studies (i.e., screening, eligibility, included in systematic review, and, if applicable, included in the meta-analysis).	6
Data collection process	10	Describe method of data extraction from reports (e.g., piloted forms, independently, in duplicate) and any processes for obtaining and confirming data from investigators.	6
Data items	11	List and define all variables for which data were sought (e.g., PICOS, funding sources) and any assumptions and simplifications made.	6
Risk of bias in individual studies	12	Describe methods used for assessing risk of bias of individual studies (including specification of whether this was done at the study or outcome level), and how this information is to be used in any data synthesis.	6
Summary measures	13	State the principal summary measures (e.g., risk ratio, difference in means).	6
Synthesis of results	14	Describe the methods of handling data and combining results of studies, if done, including measures of consistency (e.g., I^2) for each meta-analysis.	6

For peer review only - http://bmjopen.bmj.com/site/about/guidelines.xhtml

PRISMA 2009 Checklist

Section/topic	#	Checklist item	Reported on page #
Risk of bias across studies	15	Specify any assessment of risk of bias that may affect the cumulative evidence (e.g., publication bias, selective reporting within studies).	6
Additional analyses	16	Describe methods of additional analyses (e.g., sensitivity or subgroup analyses, meta-regression), if done, indicating which were pre-specified.	6
**RESULTS**			
Study selection	17	Give numbers of studies screened, assessed for eligibility, and included in the review, with reasons for exclusions at each stage, ideally with a flow diagram.	6/7
Study characteristics	18	For each study, present characteristics for which data were extracted (e.g., study size, PICOS, follow-up period) and provide the citations.	7
Risk of bias within studies	19	Present data on risk of bias of each study and, if available, any outcome level assessment (see item 12).	none
Results of individual studies	20	For all outcomes considered (benefits or harms), present, for each study: (a) simple summary data for each intervention group (b) effect estimates and confidence intervals, ideally with a forest plot.	7-12
Synthesis of results	21	Present results of each meta-analysis done, including confidence intervals and measures of consistency.	7-12
Risk of bias across studies	22	Present results of any assessment of risk of bias across studies (see Item 15).	7-12
Additional analysis	23	Give results of additional analyses, if done (e.g., sensitivity or subgroup analyses, meta-regression [see Item 16]).	7-12
**DISCUSSION**			
Summary of evidence	24	Summarize the main findings including the strength of evidence for each main outcome; consider their relevance to key groups (e.g., healthcare providers, users, and policy makers).	13-16
Limitations	25	Discuss limitations at study and outcome level (e.g., risk of bias), and at review-level (e.g., incomplete retrieval of identified research, reporting bias).	13-16
Conclusions	26	Provide a general interpretation of the results in the context of other evidence, and implications for future research.	13-16
**FUNDING**			
Funding	27	Describe sources of funding for the systematic review and other support (e.g., supply of data); role of funders for the systematic review.	17

From: Moher D, Liberati A, Tetzlaff J, Altman DG, The PRISMA Group (2009). Preferred Reporting Items for Systematic Reviews and Meta-Analyses: The PRISMA Statement. PLoS Med 6(6): e1000097. doi:10.1371/journal.pmed1000097

For more information, visit: **www.prisma-statement.org**.

For peer review only - http://bmjopen.bmj.com/site/about/guidelines.xhtml

BMJ Open

Inclusion of participants from low-and middle-income countries in Core Outcome Sets development: a systematic review

Journal:	*BMJ Open*
Manuscript ID	bmjopen-2021-049981.R1
Article Type:	Original research
Date Submitted by the Author:	31-May-2021
Complete List of Authors:	Karumbi, Jamlick; University of Liverpool, Health Data Science; KEMRI-Wellcome Trust Research Programme Nairobi, Health Systems Research<br>Gorst, Sarah L.; University of Liverpool, Department of Health Data Science<br>Gathara, David; Kenya Medical Research Institute, Wellcome Trust Research Program ; London School of Hygiene & Tropical Medicine<br>Gargon, Elizabeth; University of Liverpool, Department of Health Data Science<br>Young, Bridget; University of Liverpool, Department of Health Data Science<br>Williamson, Paula; University of Liverpool, Health Data Science
**Primary Subject Heading**:	Epidemiology
Secondary Subject Heading:	Public health, Evidence based practice
Keywords:	EPIDEMIOLOGY, PUBLIC HEALTH, Protocols & guidelines < HEALTH SERVICES ADMINISTRATION & MANAGEMENT

SCHOLARONE™
Manuscripts

BMJ

I, the Submitting Author has the right to grant and does grant on behalf of all authors of the Work (as defined in the below author licence), an exclusive licence and/or a non-exclusive licence for contributions from authors who are: i) UK Crown employees; ii) where BMJ has agreed a CC-BY licence shall apply, and/or iii) in accordance with the terms applicable for US Federal Government officers or employees acting as part of their official duties; on a worldwide, perpetual, irrevocable, royalty-free basis to BMJ Publishing Group Ltd ("BMJ") its licensees and where the relevant Journal is co-owned by BMJ to the co-owners of the Journal, to publish the Work in this journal and any other BMJ products and to exploit all rights, as set out in our licence.

The Submitting Author accepts and understands that any supply made under these terms is made by BMJ to the Submitting Author unless you are acting as an employee on behalf of your employer or a postgraduate student of an affiliated institution which is paying any applicable article publishing charge ("APC") for Open Access articles. Where the Submitting Author wishes to make the Work available on an Open Access basis (and intends to pay the relevant APC), the terms of reuse of such Open Access shall be governed by a Creative Commons licence – details of these licences and which Creative Commons licence will apply to this Work are set out in our licence referred to above.

Other than as permitted in any relevant BMJ Author's Self Archiving Policies, I confirm this Work has not been accepted for publication elsewhere, is not being considered for publication elsewhere and does not duplicate material already published. I confirm all authors consent to publication of this Work and authorise the granting of this licence.

For peer review only

Inclusion of participants from low-and middle-income countries in Core Outcome Sets development: a systematic review

Karumbi Jamlick[1,2,3*], Sarah Gorst[1], David Gathara [3,4], Elizabeth Gargon[1], Bridget Young[1], Paula Williamson[1]

[1] Department of Health Data Science, University of Liverpool

[2] Ministry of Health, Kenya

[3] Kemri-Welcome Trust Research Programme

[4] London School of Hygiene and Tropical Medicine

* Correspondence: J.Karumbi@liverpool.ac.uk

Abstract

Objective Our study aims to describe differences or similarities in the scope, participant characteristics and methods used in Core Outcome Sets (COS) development when only participants from high income countries (HICs) were involved compared to when participants from low- and middle-income countries (LMICs) were also involved.

Design Systematic review.

Data sources Annual COMET systematic reviews of COS which are updated based on SCOPUS and MEDLINE, searches. The latest systematic review included studies published up to the end of 2019.

Eligibility criteria for selecting studies We included studies reporting development of a COS for use in research regardless of age, health condition or setting. Studies reporting the development of a COS for patient reported outcomes or adverse events or complications were also included.

Data extraction and synthesis Data were extracted in relation to scope of the COS study, participant categories and the methods used in outcome selection.

Results: Studies describing 370 COS were identified in the database. Of these, 75 (20%) included participants from LMICs. Only four COS were initiated from an LMIC setting. More than half of COS with LMIC participants were developed in the last 5 years. Cancer and rheumatology were the dominant disease domains. Overall, over 259 (70%) of COS explicitly reported including clinical experts; this was higher where LMIC participants were also included 340 (92%). Most LMIC participants were from China, Brazil, and South Africa. Mixed methods for consensus building were used across the two settings.

For peer review only - http://bmjopen.bmj.com/site/about/guidelines.xhtml

Conclusion: Progress has been made in including LMIC participants in the development of COS, however there is a need to explore how to enable initiation of COS development from a range of LMIC settings, how to ensure prioritization of COS that better reflects the burden of disease in these contexts and how to improve public participation from LMICs.

For peer review only

For peer review only - http://bmjopen.bmj.com/site/about/guidelines.xhtml

Strengths and limitations of this study

- This is the first paper describing the differences and similarities in the COS development processes when there are participants from LMICs compared to when participants are exclusively from HIC settings.
- This paper describes COS published up to 2019. It is possible that more COS involving LMICs participants have been developed during 2020 or are in the process of being developed now.
- Public participation has been described in only a few studies, this may introduce a possible bias in the classification of public participants categories thereby altering the conclusions of public participation

Background

A key barrier to translating clinical research into practice is lack of conclusive evidence. This is due to different trials, of the similar interventions, being too heterogenous and reporting on outcomes that are often non comparable [1,2] or not relevant to patients and users [3,4].

Development of core outcome sets (COS) could ensure all future research in a field reports a common subset of outcomes. This would reduce research waste thereby enhancing comparability and improving research translation and use[5,6]. COS are agreed-on minimum standardized outcome sets that should be measured and reported in all research in a given health area[7]. They consist of a core domain set (this defines what domains should be measured) and core outcome measurement set (defines the instruments which would be appropriate to measure the domain)[8]. In recent years, the use of COS has been promoted by journals, Cochrane review groups, and funders[9,10]. COS have also become useful in routine clinical practice data collection for clinical audit and feedback[11,12].

Since 2010, the Core Outcome Measures in Effectiveness Trials (COMET) Initiative has been collating, stimulating and promoting the development and use of COS by maintaining an up to date publicly available searchable database[13]. The COMET database includes published studies of COS development, as well as planned and ongoing work. The types of studies included in the database are those in which COS have been developed, as well as studies relevant to COS development, including systematic reviews of outcomes. Within the database, there are publications that describe a COS only (hereafter denoted specific for COS), and other publications that describe a COS as well as provides recommendations about other trial design aspects like eligibility criteria (hereafter denoted as COS as part of a wider trial design). Studies describing the development of a patient reported outcome (PRO) COS (a core set of patients reported symptoms and health-related quality of life domains) or core event set (a core set of adverse events or complications). Initially, the database included studies

For peer review only - http://bmjopen.bmj.com/site/about/guidelines.xhtml

that had been identified by ad hoc means, however, it is now systematically updated using the annually updated systematic reviews as the basis of the updates since 2013[14–17]. The use of this database should help minimize duplication of effort in the development of COS and potentially encourage the use of COS to tackle global health challenges.

How are COS developed?

There is no gold standard for development of COS, however minimum standards have been agreed in the last few years. These are the Core Outcome Set-STAndards for Development (COS-STAD) recommendations, comprising 11 minimum standards for all COS development projects[18]. The recommendations focus on three key domains: (i) the scope of the COS; specific area of health or healthcare that the COS applies needs to be described, with details of health condition, population and types of interventions. The COS may be developed to encompass all stages or severity of a health condition or it may be focused on a particular sub-population, for example a COS may be developed for all patients with COVID-19, or it may focus on patients with long COVID only. (ii) the stakeholders involved in the COS development; the stakeholder groups to be involved and the target number from each group is dependent upon the scope of the COS and the existing knowledge and practicability. Decisions on stakeholders' involvement should be documented and explained in the study protocol. (iii) the consensus processes; this typically involves developing a consensus on 'what', 'how' and 'when' to measure. Some of the methods used to build consensus include: Delphi technique; nominal group technique, consensus development conference and semi structured group discussion. The Delphi technique with two or three rounds combined with a consensus conference has been most widely used[19–21]. Choice of method is informed by several factors including; the need to build a true consensus with methodological rigor, strategies to ensure that a diverse range of opinions are heard, and factors such as financial and carbon costs that might limit the practicality of face-to-face meetings[7].

To date, COS have been developed for various thematic or disease conditions and more are being developed[13,19]. It has been opined that development and use of a COS would help reduce outcome heterogeneity and reporting bias, while ensuring that wide-ranging perspectives, including patients' opinions, are incorporated, thus enhancing the value and quality of research[22]. Most COS have been developed from the perspectives of high-income countries (HICs), with over 70% of COS including participants from Europe and North America. There has been an increase in the proportion of COS that have included participants from

For peer review only - http://bmjopen.bmj.com/site/about/guidelines.xhtml

Africa and South American countries in COS development, but this still remains low at around 25% as per COS published up to the end of 2018 [19]. A closer look shows that the proportion of COS participants residing in low- and middle-income countries (LMICs) of these regions is even lower being just about 16% in 2016. Further, participants have mainly been those with research and clinical expertise in the various disease areas rather than as public participants with relevant lived experience [16].

Previous work describing COS studies [14–16,19] has not examined whether there is a difference in the scope, stakeholder participation and approaches to consensus between COS that have include had participants only from HICs compared to those that also included participants from LMICs. The differences in the global burden of diseases may influence which COS are developed, and available resources may determine the methods used in COS development. For example, LMICs have had a higher level of infectious diseases while non communicable diseases have been generally more prevalent in HICs. The hypothesis therefore is that COS with LMIC stakeholders are more likely to be developed for infectious disease conditions and those with predominately HIC stakeholders to are likely to be for non-communicable diseases. Additionally, different methodologies for consensus building require different level of resources, for example a face-to-face Delphi workshop may be more expensive if it must include a wider range of stakeholders and online Delphi processes are also likely to be influenced by internet connectivity which may be lower in LMICs compared to HICs. The aim of this paper is to describe the differences or similarities in the scope, participant characteristics and methods used in COS developed with participants from HICs and LMICs as classified by the Organisation for Economic Co-operation and Development (OECD)[23].

Methods

Study selection

Inclusion criteria: We included all COS studies, identified from the original COMET systematic review and annual updates[14–17,24,25]. Studies were eligible for inclusion in the review if they developed or applied methodology for determining which outcome domains or outcomes should be measured in research clinical trials or other forms of health. Studies were eligible for inclusion if they reported the development of a COS, regardless of any restrictions by age, health condition or setting. Studies describing the development of a patient reported

For peer review only - http://bmjopen.bmj.com/site/about/guidelines.xhtml

outcome (PRO) COS (a core set of patients reported symptoms and health-related quality of life domains) or core event set (a core set of adverse events or complications) were also eligible for inclusion. Studies describing the update of an existing COS are included as linked papers to the original COS[17]. Eligible studies are added to the database, as they are found with the annual update to the systematic review, ensuring the database is kept up to date.

Identification of relevant studies: The search strategy for identifying eligible COS development studies has previously been described [14–17] and is briefly described here. A comprehensive search is used to identify studies that had been published or indexed in SCOPUS and MEDLINE via Ovid from inception up to December 2019. The search strategy, which was developed for the original COMET systematic review in 2013, is provided in the appendix. Annual database searches were repeated in 2015-2020 to identify studies published up to the end of 2019. Hand searching of studies is also performed.

The review was conducted in accordance with the Preferred Reporting Items of Systematic Reviews and Meta-Analyses (PRISMA) guidelines and the included studies are shown in a PRISMA flow diagram. (see figure 1).

Data extraction:

Data were extracted in relation to scope of the COS study [aim of the study, intended use of the COS, year of publication, target population, intervention and disease or condition area], participant categories [clinicians, researchers etc, and their geographic location] and the methods used in outcome selection [Delphi, interviews, focus group discussions, etc, alone or in combination]. Data on the burden of disease in LMICs for the last five years was obtained, from the Institute of Health Metrics and Evaluation database[26], to help check its alignment to the COS developed over the same period.

Analysis:

A description of the proportion of COS that included participants from LMICs and those which were initiated from the LMIC setting is provided. For a COS to be classified as being initiated from an LMIC, either ethical approval of the study had to have been sought in an LMIC, and/or the affiliation of the lead author had to be based in an LMIC; we also used the sites of participant recruitment as confirmation. The scope, participant categories and consensus

For peer review only - http://bmjopen.bmj.com/site/about/guidelines.xhtml

methods used are described for COS that included participants from LMICs and those with exclusively HIC participants separately. Participation here is defined as being a study participant and contributing data to the COS study.

PATIENTS AND PUBLIC INVOLVEMENT

Patients and the public were not involved in the development of the research question or the design and conduct of this systematic review.

Findings

General description

There was a total of 552 references published by end of 2019 in the COMET Initiative database describing 370 COS. Of these 370 COS, 295 (80%) included only HIC participants and 75 (20%) also included LMIC participants. Two COS had only LMIC participants, one in Sri Lanka [27] and another in China [28]. A flow diagram of the study selection process is provided in Figure 1 as guided by Preferred Reporting Items for Systematic Reviews and Meta-Analyses statement[29].

Year of publication

Between 2014 and 2019, there has been an almost two-fold increase in COS that have included LMIC participants to 28% (40/141) from 15% (35/229) in preceding years.]. Figure 2 describes the absolute numbers of the COS developed up to 2019.

Scope of included studies

The comparison of the scope of the included studies is shown in Table 1. Overall, more than half (63%) of the studies were specifically designed for COS development rather than being part of a wider project or trial. However, a higher proportion of studies with LMIC participants were specific for COS (83%) compared to those with only HIC participants (58%). Over 80% of COS were developed for research only. More than half of studies (55%) did not explicitly report on the age group of the target population characteristics. During the interpretation of the data, this was assumed to be adults. This was based on the disease being predominantly associated with the adult population and the lack of reference to any literature on children.

For peer review only - http://bmjopen.bmj.com/site/about/guidelines.xhtml

Table 1 Scope of included studies

	HICs n (%) (N=295)	LMICs n (%) (N=75)	Total (%) (N=370)
**Scope of the COS study**			
**Study aims**			
Part of wider trial design#	124 (42)	13 (17)	137 (37)
Specific for COS£	171 (58)	62 (83)	233 (63)
**Intended use of recommendations**			
Research	264 (89)	61 (81)	325 (88)
Research and Practice	31 (11)	14 (19)	45 (12)
**Population characteristics**			
Neonates	4 (1)	1 (1)	5 (1)
Adults	72 (24)	16 (21)	88 (24)
Children	27 (9)	7 (9)	34 (9)
Children and Adults	28 (9)	10 (13)	38 (10)
Not specified*	164 (56)	41 (55)	205 (55)
**Intervention characteristic**			
Any intervention	77 (26)	30 (40)	107 (29)
Device	5 (2)	1 (1)	6 (2)
Device & Surgery	7 (2)	1 (1)	8 (2)
Pharmacologic Treatment	50 (17)	9 (12)	59 (16)
Procedure	9 (3)	5 (7)	14 (4)
Radiotherapy	2 (1)	0 (0)	2 (1)
Rehabilitation	9 (3)	2 (3)	11 (3)
Screening	2 (1)	0 (0)	2 (1)
Surgical	31 (11)	6 (8)	37 (10)
Vaccine	0 (0)	2 (3)	2 (3)
Both Pharmacologic & Pharmacologic treatment	3 (1)	0 (0)	3 (1)
Non-Pharmacologic Treatment	4 (1)	2 (3)	6 (2)
Health System	5 (2)	2 (3)	7 (2)
Not specified	91 (31)	15 (20)	106 (29)

#publications that describe a COS as well as provides recommendations about other trial design aspects like eligibility criteria; £ publications that describe a COS only; * *interpretated as mean adults*

Cancer (16% for COS with HIC participants only and 12% for COS with LMIC participants) and rheumatology (9% for COS with HIC participants only and 16% for COS with LMIC participants) were the dominant disease domains across the COS developed either with LMIC or HIC participants. A slightly higher proportion of COS for rheumatology, pregnancy, skin, anaesthesia, and child health included participants from LMICs compared to HICs. Five COS on health systems had LMIC participants. Figure 3 describes the disease categories that were covered by the COS.

Of the 75 COS with LMIC participants, only 5% were initiated from LMIC setting. All were from Asia, with two from China[28,30] and one each from Sri-Lanka[27] and the Philippines[31]. No COS has been initiated from Africa or South America, although COS for conditions which are more prevalent in LMIC settings had a higher number of participants from LMICs. For example, the malaria COS by Moorthy et al [32] included participants from ten sub-Sahara African Countries but none from other LMICs. The COS for the prevention of preterm births

For peer review only - http://bmjopen.bmj.com/site/about/guidelines.xhtml

had LMIC participants from Egypt, Nigeria, South Africa, China, Iran, Lebanon, Pakistan, Argentina and Brazil[33].

Characteristics of participants involved in consensus process

All COS that had LMIC participants explicitly reported characteristics of their sample compared to 92% of COS that had only HIC participants (see Table 2). Overall, over 70% of COS explicitly reported including clinical experts; this was higher where LMIC participants were involved (92%). A slightly higher proportion of COS that included LMIC participants (57%) had included members of the public compared to those with HIC participants only (32%). A higher proportion of LMIC COS (23%) had carers as part of public participants compared to the HIC COS (9%). Of the 28 COS with LMIC participants, only 4 (all with HIC patient participants too) translated the COS development materials to non-English languages. These are COS for clinical trials in acute diarrhoea [34], infant colic [35], childhood constipation [36] and type 2 diabetes[37]. Non-clinical research expertise was more common in COS with LMIC participants (44%) compared to those with HIC participants only (31%).

For peer review only

Table 2: Participant characteristics

Participant category	Sub category (not mutually exclusive)	HIC n (%#)	LMIC n (%#)	Total (%#)
**Clinical experts**		**201/295 (68)**	**69/75 (92)**	**270/370 (73)**
	Clinical experts	166 (56)	62 (83)	228 (62)
	Clinical research experts	92 (31)	41 (55)	133 (36)
	Members of a clinical trial network	8 (3)	7 (9)	15 (4)
	Clinical and non-clinical researchers	12 (4)	1 (1)	13 (4)
**Public participation**		**94/295 (32)**	**43/75 (57)**	**137/370 (37)**
	Patients	72 (24)	28 (37)	100 (27)
	Carers	26 (9)	17 (23)	43 (12)
	Patient support group representatives	21 (7)	9 (12)	30 (8)
	Service users	4 (1)	5 (7)	9 (2)
**Non-clinical research expertise**		**92/295 (31)**	**33/75 (44)**	**125/370 (34)**
	Researchers	55 (19)	21 (28)	76 (21)
	Statisticians	22 (7)	8 (11)	30 (8)
	Epidemiologists	13 (4)	9 (12)	22 (6)
	Academic representatives	5 (2)	0 (0)	5 (1)
	Methodologists	19 (6)	5 (7)	24 (6)
	Economists	7 (2)	2 (3)	9 (2)
**Authorities**		**50/295 (17)**	**22/75 (29)**	**72/370 (19)**
	Regulatory agency representatives	33 (11)	15 (20)	48 (13)
	Government agencies	14 (5)	5 (7)	19 (5)
	Policy makers	10 (3)	7 (9)	17 (5)
	Charities	4 (1)	0 (0)	4 (1)
	Service commissioners	3 (1)	1 (1)	4 (1)
**Industry representatives**		**36/295 (12)**	**19/75 (25)**	**55/270 (20)**

For peer review only - http://bmjopen.bmj.com/site/about/guidelines.xhtml

	Pharmaceutical industries	32 (11)	19 (25)	51 (14)
	Device manufacturers	4 (1)	1 (1)	5 (1)
	Biotech company representatives	1 (1)	0 (0)	1 (1)
**Others**		**10/295 (3)**	**8/75 (11)**	**18/370 (5)**
	Service providers	5 (2)	2 (3)	7 (2)
	Ethicists	1 (1)	1 (1)	2 (1)
	Journal editors	4 (1)	5 (7)	9 (2)
	Systematic review authors	13 (4)	2 (3)	15 (4)
**Not stated**	Not reported	23 (8)	0 (0)	23 (6)

[#] Since the categories are not mutually exclusive each percentage is based on the total for that category, i.e. 295 for HIC, 75 for LMIC and 370 for the Total.

Participants from LMICs were drawn from the three continents of Asia (60%), South America (49%) and Africa (31%). However, there was a skewed representation with LMIC participants largely being from three countries, with South Africa representing 50% of participants from Africa, Brazil representing 46% of participants from South America and China representing 30% of the participants from Asia.

Methods for selection of outcomes

Methods used for outcome selection ranged from Delphi, consensus conferences and semi structured discussion as shown in Table 3 below. Rarely was one method used for COS development. Semi-structured discussion seemed to be the most commonly used single method, with 20% of COS with HIC participants only and 8% of COS with LMIC participants describing its usage.

Most COS used a combination of methods. Literature review in combination with other methods was most common, with 37% of COS with HIC participants and 60% of COS with LMIC participants using this method as shown in Table 3 below. Only one COS that included LMIC participants had no methods described compared to six that only had HIC participants.

Table 3: Methods used in the selection of outcomes

Description	HIC n=295 (%)	LMIC n=75 (%)	Total n=370 (%)
Delphi only	10 (3)	5 (7)	15 (4)
Consensus conference only	11 (4)	4 (5)	15 (4)
Focus group discussions only	1 (<1)	0 (0)	1 (<1)
Nominal Group Techniques only	1 (<1)	0 (0)	1 (<1)
Semi structured discussions only	64 (22)	6 (8)	70 (19)
Survey only	4 (1)	1 (1)	5 (1)
Review of literature only	32 (4)	4 (5)	36 (10)

For peer review only - http://bmjopen.bmj.com/site/about/guidelines.xhtml

Interviews only	1 (<1)	0 (0)	1 (<1)
**Mixed methods**	**171/295 (58)**	**55/75 (73)**	**226/370 (61)**
Consensus conference and other(s)	8(3)	1 (1)	9 (2)
Delphi and other(s)	73(25)	42 (56)	115 (31)
Focus group discussions and other(s)	7 (2)	7 (9)	14 (4)
Nominal Group Techniques and other(s)	15 (5)	11 (15)	26 (7)
Semi structured discussions and other(s)	88 (30)	33 (44)	121 (33)
Survey and other(s)	25 (8)	8 (11)	33 (9)
Review of literature and other(s)	108 (37)	45 (60)	153 (41)
Interviews and other(s)	20 (7)	7 (9)	27 (7)
Unstructured and other(s)	4 (1)	8 (11)	12 (3)
No methods described	6 (2)	1 (1)	7 (2)

A combination of the three most common methods was more likely to be used for LMIC COS (n=23, 30%) compared to the HIC COS (n=37, 13%) as shown in Figure 4.

Discussion

This review provides the first comparison of COS that have been developed with participants from HICs and LMICs.

Out of the 370 published COS, 75 (20%) COS included participants from LMICs. Thirty-three COS have been published since the fifth update of COS systematic review with fifteen including participants from LMICs [19]. The COS span a range of health areas, target populations and methods used. The methods for outcome selection are similar across the settings, perhaps reflecting the increased standardization of methods of COS development generally.

Interestingly, even though participants from LMICs have been involved in earlier COS, for example, the cancer reporting recommendations by Miller and others in 1981 [38], the inclusion of LMIC participants has increased predominantly in the last 5 years. In this time, there has been an almost two-fold increase in the proportion of COS that have had LMIC participants. Global connectivity is allowing people to conduct research without the need for physical meetings. A look at the development processes used is ‘older’ COS shows that most depended on consensus building during conferences and physical workshops [32,38,39]. These kinds of consensus meetings tend to be more expensive and limit inclusion of participants from varied settings. Despite the increase, Brazil, China and South Africa have been the main drivers of participation from LMICs.

For peer review only - http://bmjopen.bmj.com/site/about/guidelines.xhtml

All the COS developed across the settings have mainly focused on research or research and practice. A slightly higher proportion (19%) of COS with LMIC participants are for research and practice compared to 11% of COS with HIC participants only. Given that COS with participants from LMICs are ‘newer’ this could reflect that more recent COS are being developed with the intention of spanning the continuum from research to routine practice in mind and also recent availability of guidance for COS development like the COMET handbook[11] and COS-STAD[18] might have influenced the inclusion of wider groups of stakeholders.

Although the COS span a wide range of conditions, the top five conditions where COS exist are cancer, rheumatology, neurology, cardiovascular and orthopaedic conditions. These are mainly non communicable diseases, and even though these are now becoming more common in LMICs, the burden of these conditions has mainly been in HICs [26]. Of the COS with LMIC participants, pregnancy, lungs and health systems were among the top five conditions. LMICs are still facing infectious diseases like neonatal sepsis, lower respiratory tract infections, malaria, and HIV among others[26,40], and one would therefore expect that COS with LMIC participation would reflect this. Only two COS, for malaria [32] and leprosy [31] represent conditions that are usually of high burden in LMICs. Thus, even though LMIC participants are being included in COS development, COS for research may not be reflecting the disease burden in LMICs. This may be because research funding priorities, for which COS are then being developed, are more likely to reflect a HIC perspective.

Interestingly, only four COS have been initiated from an LMIC setting, two from China (COS for hip-preserving treatment of osteonecrosis of the femoral head [28], COS for infertility treatment research [30]) and one each in Sri-Lanka (COS for effectiveness of antiepileptic therapy in children [27]) and the Philippines, (COS for tibialis posterior transfer surgery [31]). There is still no COS initiated from Africa or South America. Given the varying disease epidemiology and burden between HICs and LMICs there is a need to identify mechanisms to enable LMIC stakeholders to develop context relevant COS so as to improve applicability and adoptability of COS to these settings [14]. These strategies could probably include awareness raising about COS in LMIC settings, provision of resources for COS development and dissemination of guidelines and methods for COS development. These are some of the barriers which have been described potentially affecting COS development from a HIC perspective (interviewees were mainly from Europe and North America) [41]. There is a need

For peer review only - http://bmjopen.bmj.com/site/about/guidelines.xhtml

to assess which barriers and enablers are particularly relevant to COS development in LMIC settings.

Details of public participation are still sub-optimally reported, and where reported, a higher proportion of COS with LMIC participants had some public participation compared to COS with HIC participants only. Of note however, is that public participants tended to be from HIC settings. For example, the Type 2 diabetes COS included public participants from the UK and Greece[37], the breast cancer COS included breast cancer patients from US [42] and the COS for acute diarrhoea [34], infant colic [35], childhood constipation [36] all included public participants from Europe. Public participation from LMICs could potentially be improved by translation of consensus building materials into non-English languages. Harman et al, translated Delphi materials into Portuguese language in a bid to improve public participation from Brazil [37]. Unfortunately, there still were no public participants from Brazil suggesting that there are additional reasons for the lack of public participants from LMIC settings.

The lack of public participation from LMICs could be due to numerous reasons some of which could include temporal, cultural and resources differences between LMICs and HICs. First, COS that have included participants from LMICs have mostly been developed in the last decade, during which guidance has been published on how to develop COS including patient participation. Second, the availability of organized patient groups in HIC settings for given conditions or diseases provides a means of identifying and inviting patients as public participants; such groups are generally lacking in LMIC settings. Third, information asymmetry between the public and patients versus clinicians or researchers could potentially limit the participation of the former in COS development. Although evidence is scarce, we suggest that information asymmetry may be a bigger problem in LMICs due to lower literacy levels when compared to HICs [43]. Additionally, some of the major research funders in HICs, for example the National Institute for Health Research in the UK and the Patient-Centred Outcomes Research Institute in the US, are increasingly championing public participation in research.

It is therefore likely that a multipronged approach is needed. Further research to explore what would work, in addition to language translation, to improve public participation from LMICs is also needed.

Additionally, there is need to describe the stage at which public participants are involved in COS development and whether it influences which outcomes are included in the COS in a given area. Previous work has shown that patients might sometimes be inhibited when put

For peer review only - http://bmjopen.bmj.com/site/about/guidelines.xhtml

together with clinicians in a consensus building process[44] but recent experiences show this is changing, with high satisfaction reported, at least from HIC countries, where joint consensus meetings are held[37,45].

This is the first paper describing the differences and similarities in the COS development processes when there are participants from LMICs compared to when participants are exclusively from HIC settings. However, there are a few limitations. First, this paper describes COS published up to 2019. It is possible that more COS involving LMICs participants have been developed during 2020 or are in the process of being developed now. Secondly, public participation has been described in only a few studies, this may introduce a possible bias in the classification of public participants categories thereby altering the conclusions of public participation.

Conclusion

Progress has been made in including LMIC participants in the development of COS. Most COS are still being developed with a HIC perspective and as such, still reflect the priorities from HIC perspectives. Only four COS have been initiated from LMICs. Public participation is still low and is poorly documented. There is therefore, a need to explore how to enable initiation of COS development from a range of LMIC settings, how to ensure prioritization of COS that better reflects the burden of disease in these contexts and how to improve public participation from LMICs.

For peer review only

ACKNOWLEDGEMENTS

This work is also published with the permission of the Director of KEMRI.

COMPETING INTERESTS

None declared

AUTHORS CONTRIBUTIONS

The roles of the contributors were as follows: JK, DG, BY, SG, EG and PW conceptualized the review. JK screened all title and abstracts supported by PW and SG who carried out double screening of these articles. Full-text articles were screened by JK and any uncertainties were

For peer review only - http://bmjopen.bmj.com/site/about/guidelines.xhtml

discussed with the team. JK and SG extracted data. JK drafted the initial manuscript. All authors reviewed the draft manuscript and approved the final manuscript

FUNDING

JK has been supported to undertake this work as part of PhD studentship from the University of Liverpool. This studentship has no award number. The University of Liverpool had no role in developing this manuscript nor in the decision to submit for publication.

DATA SHARING STATEMENT

No additional data available. All data relevant to the study are included in the article or uploaded as supplementary information.

ETHICS STATEMENT

Ethical approval was not required for this study.

For peer review only

For peer review only - http://bmjopen.bmj.com/site/about/guidelines.xhtml

References

[1] Heneghan C, Goldacre B, Mahtani KR. Why clinical trial outcomes fail to translate into benefits for patients. Trials 2017;18:122. https://doi.org/10.1186/s13063-017-1870-2.

[2] Duffy JMN, Ziebland S, von Dadelszen P, McManus RJ. Tackling poorly selected, collected, and reported outcomes in obstetrics and gynecology research. Am J Obstet Gynecol 2019;220:71.e1-71.e4. https://doi.org/10.1016/j.ajog.2018.09.023.

[3] Sinha IP, Williamson PR, Smyth RL. Outcomes in Clinical Trials of Inhaled Corticosteroids for Children with Asthma Are Narrowly Focussed on Short Term Disease Activity. PLoS One 2009;4:e6276.

[4] Tong A, Crowe S, Gill JS, Harris T, Hemmelgarn BR, Manns B, et al. Clinicians' and researchers' perspectives on establishing and implementing core outcomes in haemodialysis: semistructured interview study. BMJ Open 2018;8:e021198. https://doi.org/10.1136/bmjopen-2017-021198.

[5] Clarke M. Standardising outcomes for clinical trials and systematic reviews. Trials 2007;8:1–3. https://doi.org/10.1186/1745-6215-8-39.

[6] Chalmers I, Glasziou P. Avoidable waste in the production and reporting of research evidence. Lancet (London, England) 2009;374:86–9. https://doi.org/10.1016/S0140-6736(09)60329-9.

[7] Williamson PR, Altman DG, Blazeby JM, Clarke M, Devane D, Gargon E, et al. Developing core outcome sets for clinical trials: issues to consider. Trials 2012;13:132. https://doi.org/10.1186/1745-6215-13-132.

[8] Chevance A, Tran VT, Ravaud P. Improving the generalizability and credibility of core outcome sets (COSs) by a large and international participation of diverse stakeholders. J Clin Epidemiol 2020:1–8. https://doi.org/10.1016/j.jclinepi.2020.01.004.

[9] Hughes KL, Kirkham JJ, Clarke M, Williamson PR. Assessing the impact of a research funder's recommendation to consider core outcome sets. PLoS One 2019;14:e0222418–e0222418. https://doi.org/10.1371/journal.pone.0222418.

[10] COMET Initiative. COS endorsments 2020. http://www.comet-initiative.org/COSEndorsement (accessed September 26, 2020).

[11] Williamson PR, Altman DG, Bagley H, Barnes KL, Blazeby JM, Brookes ST, et al. The COMET Handbook: version 1.0. Trials 2017;18:280. https://doi.org/10.1186/s13063-017-1978-4.

[12] Webbe JWH, Duffy JMN, Afonso E, Al-Muzaffar I, Brunton G, Greenough A, et al. Core outcomes in neonatology: development of a core outcome set for neonatal research. Arch Dis Child - Fetal Neonatal Ed 2019:fetalneonatal-2019-317501. https://doi.org/10.1136/archdischild-2019-317501.

[13] COMET Initiative. COMET_database n.d. http://www.comet-initiative.org/Studies (accessed February 10, 2020).

[14] Gargon E, Gurung B, Medley N, Altman DG, Blazeby JM, Clarke M, et al. Choosing important health outcomes for comparative effectiveness research: A systematic review. PLoS One 2014;9. https://doi.org/10.1371/journal.pone.0099111.

[15] Gorst SL, Gargon E, Clarke M, Blazeby JM, Altman DG, Williamson PR. Choosing important health outcomes for comparative effectiveness research: An updated review and user survey. PLoS One 2016;11:1–12. https://doi.org/10.1371/journal.pone.0146444.

[16] Davis K, Gorst SL, Harman N, Smith V, Gargon E, Altman DG, et al. Choosing important health outcomes for comparative effectiveness research: An updated systematic review and involvement of low and middle income countries. PLoS One 2018;13:1–14. https://doi.org/10.1371/journal.pone.0190695.

[17] Gargon E, Gorst SL, Williamson PR. Choosing important health outcomes for comparative effectiveness research: 5th annual update to a systematic review of core outcome sets for research. PLoS One 2019;14:1–18. https://doi.org/10.1371/journal.pone.0225980.

[18] Kirkham JJ, Davis K, Altman DG, Blazeby JM, Clarke M, Tunis S, et al. Core Outcome Set-STAndards for Development: The COS-STAD recommendations. PLoS Med

For peer review only

For peer review only - http://bmjopen.bmj.com/site/about/guidelines.xhtml

2017;14:e1002447–e1002447. https://doi.org/10.1371/journal.pmed.1002447.
[19] Gargon E, Gorst SL, Williamson PR. Choosing important health outcomes for comparative effectiveness research: 5th annual update to a systematic review of core outcome sets for research. PLoS One 2019;14:e0225980–e0225980. https://doi.org/10.1371/journal.pone.0225980.
[20] Sinha IP, Smyth RL, Williamson PR. Using the Delphi technique to determine which outcomes to measure in clinical trials: recommendations for the future based on a systematic review of existing studies. PLoS Med 2011;8:e1000393–e1000393. https://doi.org/10.1371/journal.pmed.1000393.
[21] Turnbull AE, Dinglas VD, Friedman LA, Chessare CM, Sepúlveda KA, Bingham III CO, et al. A survey of Delphi panelists after core outcome set development revealed positive feedback and methods to facilitate panel member participation. J Clin Epidemiol 2018;102:99–106. https://doi.org/10.1016/j.jclinepi.2018.06.007.
[22] Williamson P, Altman D, Blazeby J, Clarke M, Gargon E. Driving up the Quality and Relevance of Research Through the Use of Agreed Core Outcomes. J Health Serv Res Policy 2012;17:1–2. https://doi.org/10.1258/jhsrp.2011.011131.
[23] OECD. Development Assistance Committee List of Official Development Assistance Recipients 2013. http://www.oecd.org/dac/stats/49483614.pdf.
[24] Gorst SL, Gargon E, Clarke M, Smith V, Williamson PR. Choosing important health outcomes for comparative effectiveness research: An updated review and identification of gaps. PLoS One 2016;11:1–14. https://doi.org/10.1371/journal.pone.0168403.
[25] Gargon E, Gorst SL, Harman NL, Smith V, Matvienko-Sikar K, Williamson PR. Choosing important health outcomes for comparative effectiveness research: 4th annual update to a systematic review of core outcome sets for research. PLoS One 2018;13:1–15. https://doi.org/10.1371/journal.pone.0209869.
[26] Institute of Health Metrics and Evaluation. Global Burden of Disease 2020. https://vizhub.healthdata.org/gbd-compare/ (accessed December 1, 2020).
[27] Murugupillai R, Ranganathan SS, Wanigasinghe J, Muniyandi R, Arambepola C. Development of outcome criteria to measure effectiveness of antiepileptic therapy in children. Epilepsy Behav 2018;80:56–60. https://doi.org/10.1016/j.yebeh.2017.12.011.
[28] Xue Z, Sun J, Li T, Huang Z, Chen W. How to evaluate the clinical outcome of joint-preserving treatment for osteonecrosis of the femoral head: development of a core outcome set. J Orthop Surg Res 2019;14:317. https://doi.org/10.1186/s13018-019-1364-x.
[29] Moher D, Liberati A, Tetzlaff J, Altman DG, Group TP. Preferred Reporting Items for Systematic Reviews and Meta-Analyses: The PRISMA Statement. PLOS Med 2009;6:e1000097.
[30] Legro RS, Wu X, Barnhart KT, Farquhar C, Fauser BCJM, Mol B. Improving the reporting of Clinical trials of infertility treatments (IMPRINT): Modifying the CONSORT statement. Hum Reprod 2014;29:2075–82. https://doi.org/10.1093/humrep/deu218.
[31] Cross H. A Delphi consensus on criteria for contraindications, assessment indicators and expected outcomes related to tibialis posterior transfer surgery. Int J Lepr Other Mycobact Dis Off Organ Int Lepr Assoc 2005;73:13–21. https://doi.org/10.1489/1544-581X(2005)73[13:ADCOCF]2.0.CO;2.
[32] Moorthy V, Reed Z, Smith PG. Measurement of malaria vaccine efficacy in phase III trials: Report of a WHO consultation. Vaccine 2007;25:5115–23. https://doi.org/10.1016/j.vaccine.2007.01.085.
[33] Van't Hooft J, Duffy JMN, Daly M, Williamson PR, Meher S, Thom E, et al. A core outcome set for evaluation of interventions to prevent preterm birth. Obstet Gynecol 2016;127:49–58. https://doi.org/10.1097/AOG.0000000000001195.
[34] Karas J, Ashkenazi S, Guarino A, Vecchio A Lo, Shamir R, Vandenplas Y, et al. A core outcome set for clinical trials in acute diarrhoea. Arch Dis Child 2015;100:359–63. https://doi.org/10.1136/archdischild-2014-307403.

For peer review only - http://bmjopen.bmj.com/site/about/guidelines.xhtml

[35] Steutel NF, Benninga MA, Langendam MW, Korterink JJ, Indrio F, Szajewska H, et al. Developing a core outcome set for infant colic for primary, secondary and tertiary care settings: A prospective study. BMJ Open 2017;7:1–11. https://doi.org/10.1136/bmjopen-2016-015418.

[36] Kuizenga-Wessel S, Steutel NF, Benninga MA, Devreker T, Scarpato E, Staiano A, et al. Development of a core outcome set for clinical trials in childhood constipation: a study using a Delphi technique. BMJ Paediatr Open 2017;1:e000017. https://doi.org/10.1136/bmjpo-2017-000017.

[37] Harman NL, Wilding JPH, Curry D, Harris J, Logue J, Pemberton RJ, et al. Selecting Core Outcomes for Randomised Effectiveness trials In Type 2 diabetes (SCORE-IT): a patient and healthcare professional consensus on a core outcome set for type 2 diabetes. BMJ Open Diabetes Res &Amp; Care 2019;7:e000700. https://doi.org/10.1136/bmjdrc-2019-000700.

[38] Miller A, Hoogstraten B, Staquet M, Winkler A. Reporting Results of Cancer Trials. Cancer 1981;47:207–14.

[39] Heijde V der D, Bellamy N, Calin A. Preliminary Core Sets for Endpoints in Ankylosing Spondilitis. J Rheumatol 1997;24:2225–9.

[40] Vos T, Lim SS, Abbafati C, Abbas KM, Abbasi M, Abbasifard M, et al. Global burden of 369 diseases and injuries in 204 countries and territories, 1990–2019: a systematic analysis for the Global Burden of Disease Study 2019. Lancet 2020;396:1204–22. https://doi.org/10.1016/S0140-6736(20)30925-9.

[41] Gargon E, Williamson PR, Young B. Improving core outcome set development: qualitative interviews with developers provided pointers to inform guidance. J Clin Epidemiol 2017;86:140–52. https://doi.org/10.1016/j.jclinepi.2017.04.024.

[42] Ong WL, Schouwenburg MG, Van Bommel ACM, Stowell C, Allison KH, Benn KE, et al. A standard set of value-based patient-centered outcomes for breast cancer: The International Consortium for Health Outcomes Measurement (ICHOM) initiative. JAMA Oncol 2017;3:677–85. https://doi.org/10.1001/jamaoncol.2016.4851.

[43] Bloom G, Standing H, Lloyd R. Markets, information asymmetry and health care: Towards new social contracts. Soc Sci Med 2008;66:2076–87. https://doi.org/10.1016/j.socscimed.2008.01.034.

[44] Young B, Bagley H. Including patients in core outcome set development: issues to consider based on three workshops with around 100 international delegates. Res Involv Engagem 2016;2:25. https://doi.org/10.1186/s40900-016-0039-6.

[45] Hall DA, Smith H, Hibbert A, Colley V, Haider HF, Horobin A, et al. The COMiT'ID Study: Developing Core Outcome Domains Sets for Clinical Trials of Sound-, Psychology-, and Pharmacology-Based Interventions for Chronic Subjective Tinnitus in Adults. Trends Hear 2018;22:1–16. https://doi.org/10.1177/2331216518814384.

For peer review only

List of figures

Figure 1 Preferred Reporting Items for Systematic Reviews and Meta-Analyses flowchart of identification of eligible studies from the COMET database. Data were extracted from the COS systematic reviews [14–17]. 5

Figure 2 Year of COS publication 6

Figure 3 Comparison of disease categories covered in the COS with exclusively HIC participants and those also with LMIC participants 7

Figure 4 Combinations of methods for outcome selection 9

Appendix: Search strategy 6

For peer review only - http://bmjopen.bmj.com/site/about/guidelines.xhtml

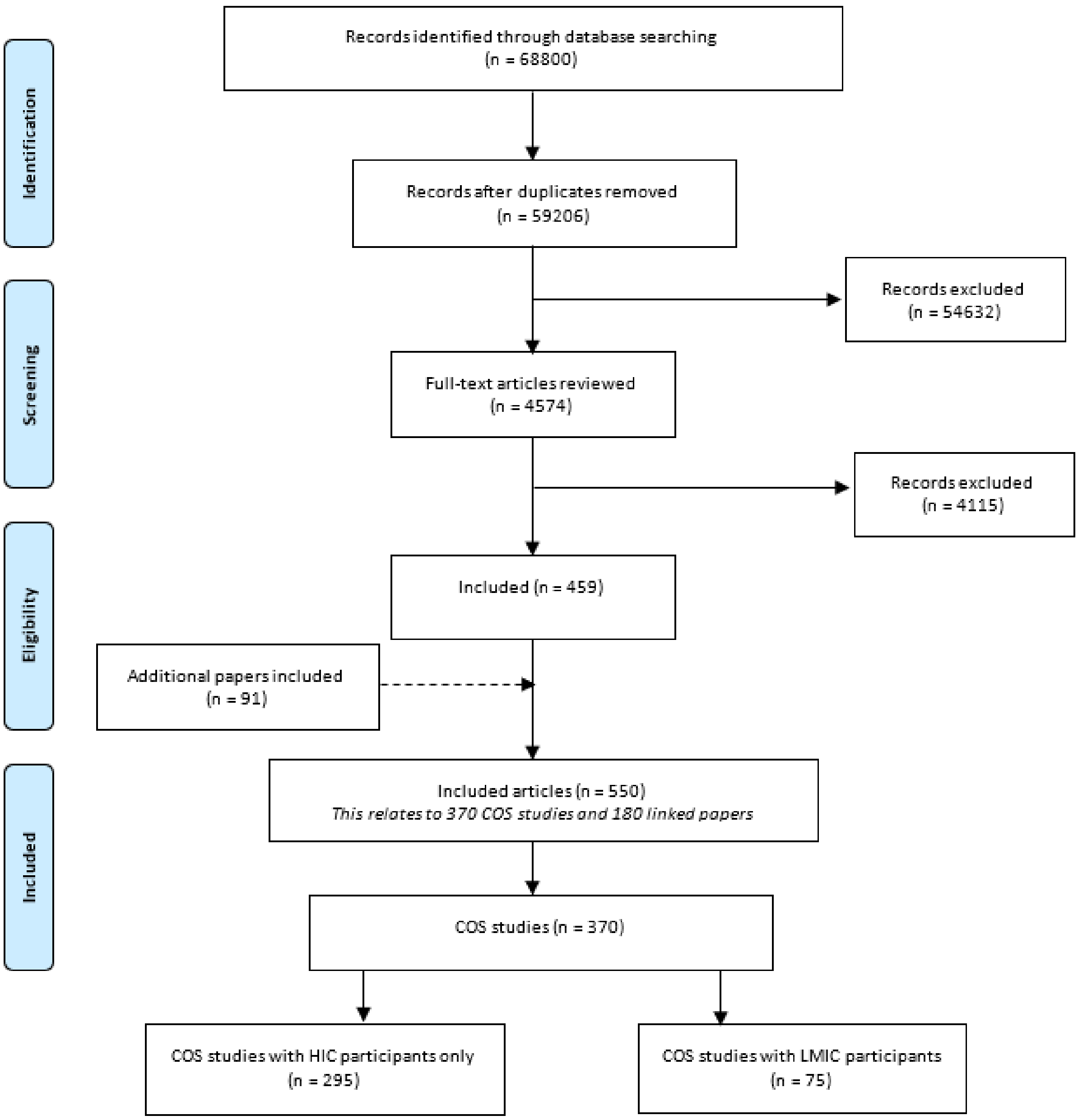

Figure 1 Preferred Reporting Items for Systematic Reviews and Meta-Analyses flowchart of identification of eligible studies from the COMET database. Data were extracted from the COS systematic reviews [14–17].

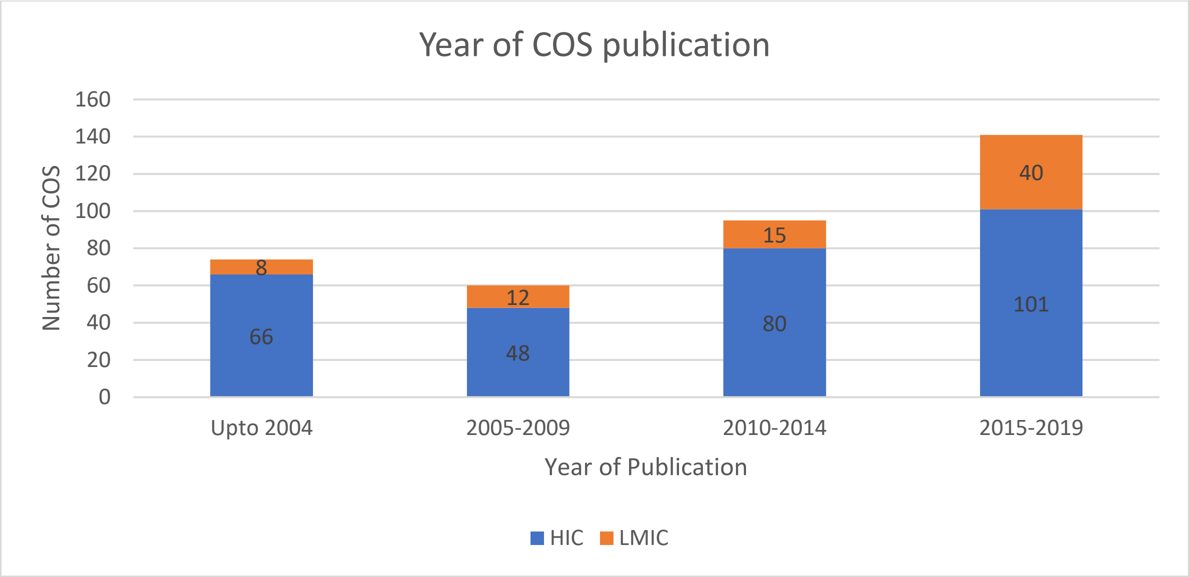

Figure 2 Year of COS publication

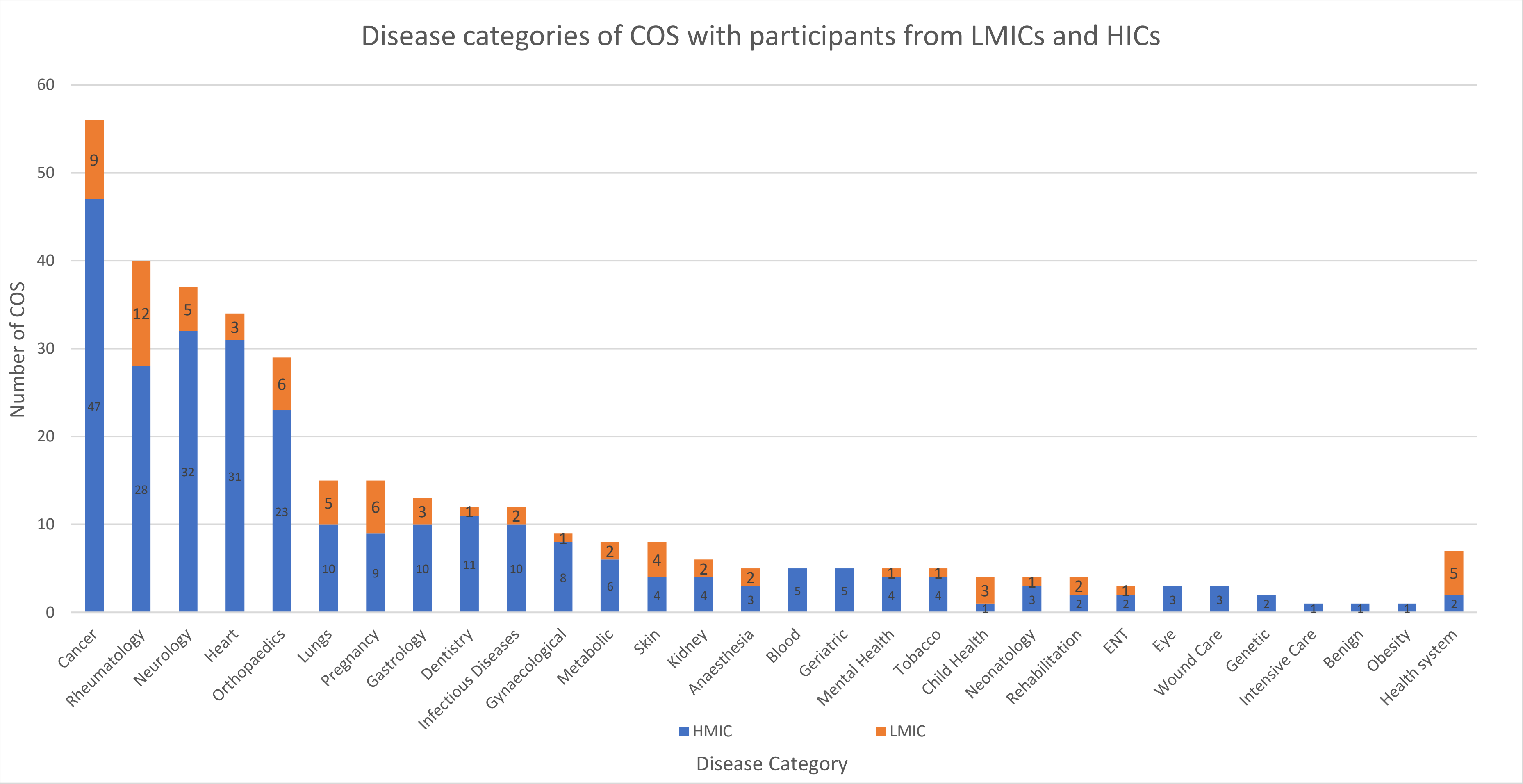

Figure 3 Comparison of disease categories covered in the COS with exclusively HIC participants and those also with LMIC participants

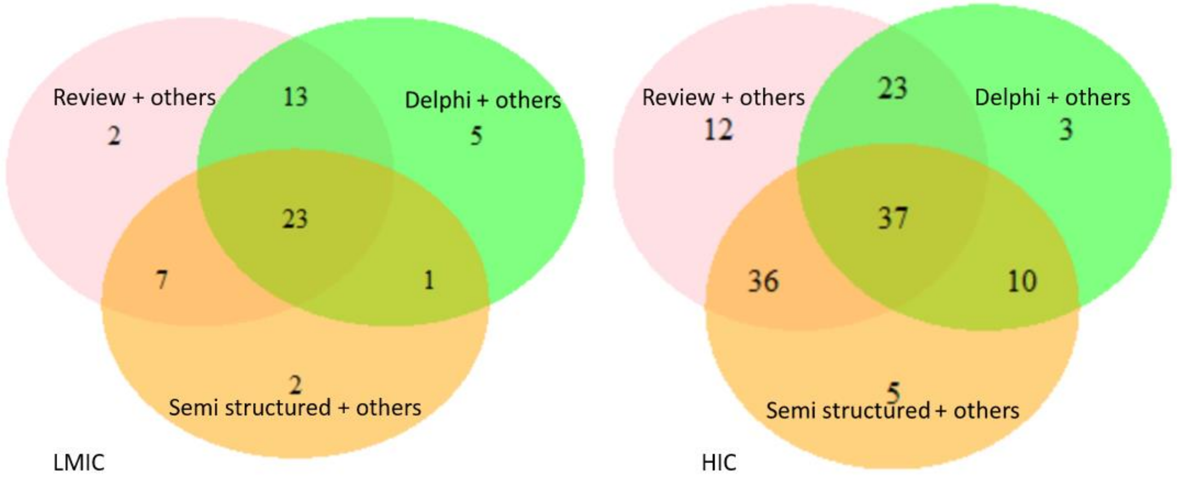

Figure 4 Methods used in COS development across LMIC and HIC

For peer review only

Appendix A. Search strategy (As described by Gorst et al [1]

**Search terms for MEDLINE**	
	**Randomised trial and systematic review terms**
1	Health Services/ut [Utilization]
2	registries/
3	systematic review.mp.
4	structured review.ti.
5	evidence based medicine.ab.
6	exp Clinical Trials as Topic/
7	clinical trial$.ab.
8	randomised controlled trial$.ti,ab.
9	randomised trial$.ti,ab.
10	1 or 2 or 3 or 4 or 5 or 6 or 7 or 8 or 9
	**Methodology terms**
11	workgroup$.mp.
12	standard$ outcome$.mp.
13	Practice Guideline/
14	clinical database.mp.
15	patient important outcome$.mp.
16	(standard$ adj3 reporting).mp.
17	congresses.pt.
18	Delphi Technique/
19	(recommend$ adj3 outcome$).mp.
20	consensus development conference.pt.
21	outcome$ reporting.mp.
22	priorit$ symptom$.mp.
23	(task force adj3 outcome$).mp.
24	appropriate outcome$.mp.
25	research design/
26	endpoint determination/
27	consensus development conference/
28	patient participation/
29	consensus.mp.
30	workshop.mp.
31	Consensus Development Conferences, NIH as Topic/
32	focus groups/
33	11 or 12 or 13 or 14 or 15 or 16 or 17 or 18 or 19 or 20 or 21 or 22 or 23 or 24 or 25 or 26 or 27 or 28 or 29 or 30 or 31 or 32
	**Outcome terms**
34	outcome$.mp.

For peer review only - http://bmjopen.bmj.com/site/about/guidelines.xhtml

35	end point$.mp.
36	(core adj3 set).mp.
37	treatment emergent problem$.mp.
38	exp outcome Assessment Health Care/
39	Treatment Outcome/
40	Quality of Life/
41	34 or 35 or 36 or 37 or 38 or 39 or 40
	**Key terms targeted**
42	clinical-study design.mp.
43	patient$ perspective$.ti.
44	outcome$.mp. and delphi.ti.
45	(outcome$ and delphi).ab.
46	(perspective$ adj3 outcome$).ti.
47	core outcome$.ti,ab.
48	core set$.ti,ab.
49	clinical trial design$.ti.
50	design$ clinical trial$.ti.
51	(consensus and outcome$).ti.
52	42 or 43 or 44 or 45 or 46 or 47 or 48 or 49 or 50 or 51
53	10 and 33 and 41
54	52 or 53

**Search terms for SCOPUS**
((((INDEXTERMS(registries)) OR (INDEXTERMS(clinical trials as topic)) OR (ABS("evidence based medicine")) OR (ABS("clinical trial*")) OR (INDEXTERMS("Health Services Utilization")) OR (TITLE-ABS-KEY("SYSTEMATIC REVIEW")) OR (TITLE("structured review"))) OR (TITLE OR ABS("randomised controlled trial*")) OR (TITLE OR ABS (randomised trial*))) AND (((TITLE-ABS-KEY(workgroup*)) OR (TITLE-ABS-KEY(standard* outcome*)) OR (INDEXTERMS(practice guideline)) OR (TITLE-ABS-KEY("clinical database")) OR (TITLE-ABS-KEY("patient important outcome*")) OR (TITLE-ABS-KEY("standard* outcome*")) OR (INDEXTERMS(delphi technique))) OR ((TITLE-ABS-KEY(recommend* W/3 outcome*)) OR (TITLE-ABS-KEY(standard* W/3 reporting*)) OR (TITLE-ABS-KEY(task force W/3 outcome*)) OR (TITLE-ABS-KEY("appropriate outcome*")) OR (TITLE-ABS-KEY("outcome* reporting")) OR (TITLE-ABS-KEY("priorit* symptom*")) OR (INDEXTERMS(focus group)) (INDEXTERMS(research design))) OR ((INDEXTERMS(endpoint determination)) OR (INDEXTERMS(consensus development conference)) OR (INDEXTERMS(patient participation)) OR (TITLE-ABS-KEY(consensus)) OR (TITLE-ABS-KEY(workshop)))) AND 74) OR (((TITLE("design* clinical trials")) OR (TITLE(consensus AND outcome*)) OR (TITLE-ABS-KEY("clinical-study design")) OR (TITLE("patient* perspective*")) OR (ABS(outcome* AND delphi)) OR (TITLE(outcome* AND delphi)) OR (TITLE(perspective* W/3 outcome*)) OR (ABS("core outcome*") OR TITLE("core outcome*"))) OR ((ABS("core set*") OR TITLE("core set*")) OR (TITLE("clinical trial design*"))))

[1] Gorst SL, Prinsen CAC, Salcher-Konrad M, Matvienko-Sikar K, Williamson PR, Terwee CB. Methods used in the selection of instruments for outcomes included in core outcome sets have improved since the publication of the COSMIN/COMET guideline. J Clin Epidemiol 2020;125:64–75. https://doi.org/https://doi.org/10.1016/j.jclinepi.2020.05.021.

For peer review only - http://bmjopen.bmj.com/site/about/guidelines.xhtml

PRISMA 2009 Checklist

Section/topic	#	Checklist item	Reported on page #
**TITLE**			
Title	1	Identify the report as a systematic review, meta-analysis, or both.	1
**ABSTRACT**			
Structured summary	2	Provide a structured summary including, as applicable: background; objectives; data sources; study eligibility criteria, participants, and interventions; study appraisal and synthesis methods; results; limitations; conclusions and implications of key findings; systematic review registration number.	1
**INTRODUCTION**			
Rationale	3	Describe the rationale for the review in the context of what is already known.	5
Objectives	4	Provide an explicit statement of questions being addressed with reference to participants, interventions, comparisons, outcomes, and study design (PICOS).	5
**METHODS**			
Protocol and registration	5	Indicate if a review protocol exists, if and where it can be accessed (e.g., Web address), and, if available, provide registration information including registration number.	none
Eligibility criteria	6	Specify study characteristics (e.g., PICOS, length of follow-up) and report characteristics (e.g., years considered, language, publication status) used as criteria for eligibility, giving rationale.	5-6
Information sources	7	Describe all information sources (e.g., databases with dates of coverage, contact with study authors to identify additional studies) in the search and date last searched.	6
Search	8	Present full electronic search strategy for at least one database, including any limits used, such that it could be repeated.	Appendix
Study selection	9	State the process for selecting studies (i.e., screening, eligibility, included in systematic review, and, if applicable, included in the meta-analysis).	6
Data collection process	10	Describe method of data extraction from reports (e.g., piloted forms, independently, in duplicate) and any processes for obtaining and confirming data from investigators.	6
Data items	11	List and define all variables for which data were sought (e.g., PICOS, funding sources) and any assumptions and simplifications made.	6
Risk of bias in individual studies	12	Describe methods used for assessing risk of bias of individual studies (including specification of whether this was done at the study or outcome level), and how this information is to be used in any data synthesis.	none
Summary measures	13	State the principal summary measures (e.g., risk ratio, difference in means).	6
Synthesis of results	14	Describe the methods of handling data and combining results of studies, if done, including measures of consistency (e.g., I^2) for each meta-analysis.	None

For peer review only - http://bmjopen.bmj.com/site/about/guidelines.xhtml

PRISMA 2009 Checklist

Section/topic	#	Checklist item	Reported on page #
Risk of bias across studies	15	Specify any assessment of risk of bias that may affect the cumulative evidence (e.g., publication bias, selective reporting within studies).	None
Additional analyses	16	Describe methods of additional analyses (e.g., sensitivity or subgroup analyses, meta-regression), if done, indicating which were pre-specified.	None
**RESULTS**			
Study selection	17	Give numbers of studies screened, assessed for eligibility, and included in the review, with reasons for exclusions at each stage, ideally with a flow diagram.	7
Study characteristics	18	For each study, present characteristics for which data were extracted (e.g., study size, PICOS, follow-up period) and provide the citations.	7
Risk of bias within studies	19	Present data on risk of bias of each study and, if available, any outcome level assessment (see item 12).	None
Results of individual studies	20	For all outcomes considered (benefits or harms), present, for each study: (a) simple summary data for each intervention group (b) effect estimates and confidence intervals, ideally with a forest plot.	7-11
Synthesis of results	21	Present results of each meta-analysis done, including confidence intervals and measures of consistency.	None
Risk of bias across studies	22	Present results of any assessment of risk of bias across studies (see Item 15).	None
Additional analysis	23	Give results of additional analyses, if done (e.g., sensitivity or subgroup analyses, meta-regression [see Item 16]).	None
**DISCUSSION**			
Summary of evidence	24	Summarize the main findings including the strength of evidence for each main outcome; consider their relevance to key groups (e.g., healthcare providers, users, and policy makers).	11-14
Limitations	25	Discuss limitations at study and outcome level (e.g., risk of bias), and at review-level (e.g., incomplete retrieval of identified research, reporting bias).	14
Conclusions	26	Provide a general interpretation of the results in the context of other evidence, and implications for future research.	11-14
**FUNDING**			
Funding	27	Describe sources of funding for the systematic review and other support (e.g., supply of data); role of funders for the systematic review.	15

From: Moher D, Liberati A, Tetzlaff J, Altman DG, The PRISMA Group (2009). Preferred Reporting Items for Systematic Reviews and Meta-Analyses: The PRISMA Statement. PLoS Med 6(6): e1000097. doi:10.1371/journal.pmed1000097

For more information, visit: www.prisma-statement.org.

For peer review only - http://bmjopen.bmj.com/site/about/guidelines.xhtml
